# SGLT5 is the renal transporter for 1,5-anhydroglucitol, a major player in two rare forms of neutropenia

**DOI:** 10.1007/s00018-023-04884-8

**Published:** 2023-08-18

**Authors:** Jennifer Diederich, Pierre Mounkoro, Hernan A. Tirado, Nathalie Chevalier, Emile Van Schaftingen, Maria Veiga-da-Cunha

**Affiliations:** Metabolic Research Group, de Duve Institute and UCLouvain, de Duve Institute, 75, Av. Hippocrate, 1200 Brussels, Belgium

**Keywords:** 1,5-Anhydroglucitol, SGLT5, SGLT4, GSD1b, Neutropenia, G6PC3-deficiency, Empagliflozin, SGLT2-inhibitors, Glycogen storage disease type Ib, SCN4

## Abstract

Neutropenia and neutrophil dysfunction in glycogen storage disease type 1b (GSD1b) and severe congenital neutropenia type 4 (SCN4), associated with deficiencies of the glucose-6-phosphate transporter (G6PT/SLC37A4) and the phosphatase G6PC3, respectively, are the result of the accumulation of 1,5-anhydroglucitol-6-phosphate in neutrophils. This is an inhibitor of hexokinase made from 1,5-anhydroglucitol (1,5-AG), an abundant polyol in blood. 1,5-AG is presumed to be reabsorbed in the kidney by a sodium-dependent-transporter of uncertain identity, possibly SGLT4/*SLC5A9* or SGLT5/*SLC5A10*. Lowering blood 1,5-AG with an SGLT2-inhibitor greatly improved neutrophil counts and function in G6PC3-deficient and GSD1b patients. Yet, this effect is most likely mediated indirectly, through the inhibition of the renal 1,5-AG transporter by glucose, when its concentration rises in the renal tubule following inhibition of SGLT2. To identify the 1,5-AG transporter, both human and mouse SGLT4 and SGLT5 were expressed in HEK293T cells and transport measurements were performed with radiolabelled compounds. We found that SGLT5 is a better carrier for 1,5-AG than for mannose, while the opposite is true for human SGLT4. Heterozygous variants in SGLT5, associated with a low level of blood 1,5-AG in humans cause a 50–100% reduction in 1,5-AG transport activity tested in model cell lines, indicating that SGLT5 is the predominant kidney 1,5-AG transporter. These and other findings led to the conclusion that (1) SGLT5 is the main renal transporter of 1,5-AG; (2) frequent heterozygous mutations (allelic frequency > 1%) in SGLT5 lower blood 1,5-AG, favourably influencing neutropenia in G6PC3 or G6PT deficiency; (3) the effect of SGLT2-inhibitors on blood 1,5-AG level is largely indirect; (4) specific SGLT5-inhibitors would be more efficient to treat these neutropenias than SGLT2-inhibitors.

**Supplementary Information:**

The online version contains supplementary material available at 10.1007/s00018-023-04884-8.

## Introduction

1,5-anhydroglucitol (1,5-AG), also known as 1-deoxyglucose, is a six-carbon monosaccharide similar to glucose and the most abundant polyol present in blood [1]. We have recently shown that its presence in blood is at the origin of the neutropenia in G6PC3-deficient and glycogen storage disease type 1b (GSD1b) patients [2–4]. Once in neutrophils, 1,5-AG is slowly phosphorylated to 1,5-anhydroglucitol-6-phosphate (1,5-AG6P) by a side activity of low-Km hexokinases and ADPGK. Accumulation of 1,5-AG6P is normally prevented by the phosphatase G6PC3, which dephosphorylates 1,5-AG6P inside the endoplasmic reticulum, following its transport into this cell compartment by the glucose-6-phosphate transporter (SLC37A4/G6PT). By contrast, the neutrophils of glucose-6-phosphatase catalytic subunit 3 (G6PC3)-deficient and GSD1b (defect in G6PT) patients, accumulate 1,5-AG6P in their neutrophils to concentrations that inhibit low-Km hexokinases. This slows down glycolysis, NADPH production in the pentose-phosphate pathway (required for an efficient respiratory burst) and impairs protein glycosylation  causing the neutropenia and neutrophil dysfunction observed in these patients [2, 5, 6].

1,5-AG comes largely from food (90%) and to a lesser extent from endogenous synthesis [7]. Despite no ascribed physiological role, after being freely filtered by the kidney, 1,5-AG is almost entirely reabsorbed by an active transporter present in the proximal renal tubule, instead of being excreted in the urine. It is the active reabsorption of 1,5-AG and the observation that its metabolism is negligible, that accounts for the fact that the concentration of 1,5-AG remains stable in the body and in blood [8, 9]. However, when glucose concentration rises above the renal threshold for glucose, typically > 160–180 mg/dL (8.9–10 mmol/L) [7, 10], as is the case in diabetes, the excess glucose competes with 1,5-AG for reabsorption in the proximal tubule. In turn, this increases the urinary excretion of 1,5-AG and explains the decrease in the concentration of 1,5-AG in blood that is seen in uncontrolled diabetic individuals [10–12] or in controlled diabetics that are treated with gliflozins, a new class of antidiabetic molecules acting as sodium-glucose cotransporter-2 (SGLT2)-inhibitors [13]. Inspired by this observation, we have recently repurposed empagliflozin, one of the available SGLT2-inhibitors, to treat neutropenia and neutrophil dysfunction in GSD1b and G6PC3-deficient patients. We and others [3, 4, 14–17] have shown that by increasing the concentration of glucose in the urinary filtrate, SGLT2-inhibitors safely lower 1,5-AG in blood and as a result are now becoming widely used in the clinic to treat these patients’ neutropenia.

Yet, the identity of the renal transporter(s) of 1,5-AG remains to be established and the evidence for it being *SLC5A9*/SGLT4, *SLC5A10*/SGLT5 or both is still conflicting. SGLT4 is often referred to in the literature [18–20] as the renal 1,5-AG transporter, but the evidence is mainly based on transport experiments tested with a non-physiological (30 mM) concentration of 1,5-AG [18] in transiently transfected COS-7 cells overexpressing human SGLT4. On the other hand, rare SNPs in SGLT5 [21–23] are associated with lower 1,5-AG in blood, suggesting that the renal transporter of 1,5-AG could be SGLT5, but this has never been functionally shown. Furthermore, we have recently found the presence of a rare SNP in SGLT5 (p.Arg401His) that is predicted to damage protein function, in a G6PC3-deficient patient with a high urinary clearance of 1,5-AG [4].

To untangle this question, and in the hope of further improving the treatment of these two genetically inherited forms of neutropenia, we characterize in depth the ability of human and mouse SGLT4 and SGLT5 to transport 1,5-AG or other sugars and show the functional impact of the rare SNPs found in SGLT5 on the transport of 1,5-AG.

## Materials and methods

### Materials

D-U-[^14^C]-mannose was obtained from American Radiolabeled Chemicals—ARC (St. Louis, Missouri, USA). 2-[^3^H]-1,5-anhydroglucitol was prepared in house by reduction of 1,5-anhydrofructose with sodium-[^3^H]-borohydride as described [2]. 1,5-anhydroglucitol, 1,5-anhydromannitol and 1,5-anhydrofructose were from Biosynth (Bratislava, Slovakia). Other hexoses, empagliflozin and poly-d-lysine were from Sigma–Aldrich (St. Louis, Missouri, USA). Cytochalasin B was purchased from Fermentek Ltd (Jerusalem, Israel). Remogliflozin and dapagliflozin were from Axon Medchem (Groningen, The Netherlands). HEK293T cells were cultured in high glucose Dulbecco’s modified Eagle’s medium (DMEM) without glutamine from Biowest (Nuaillé, France) supplemented with 10% heat-inactivated FBS, 100 U/mL penicillin, 100 μg/mL streptomycin, 1 mM sodium pyruvate, and 1 × non-essential amino acids (MEM) solution. Puromycin dihydrochloride (1.5 µg/ml, Fisher BioReagents™) was added to the culture medium for selection when needed.

### Cloning and transient expression in HEK293T cells of recombinant mouse and human SGLT4 and SGLT5

To overexpress SGLT4 and SGLT5, we chose HEK293T cells, which spontaneously do not express SGLT4 or SGLT5 (as indicated in Human Protein Atlas database). Furthermore, HEK293 derived cells have previously been used by Grempler et al. [24] to study the properties of SGLT5. The coding sequences of human and mouse SGLT4 (NM_001011547; NM_145551) and SGLT5 (NM_001011547; NM_001033227) were PCR amplified from cDNA prepared from human or mouse intestine (SGLT4) and kidney (SGLT5) by reverse transcription using Revertaid Reverse Transcriptase (Thermo Scientific) according to the manufacturers’ instructions. Mouse mRNA was isolated in the lab and human mRNA was from Human RNA master panel II (Clontech). The PCR-amplified sequences were inserted in vectors pEF6/Myc-HisA or pEF6/HisB, which allow the addition of a C-terminal or a N-terminal 6xHis-tag. Plasmids for expression of untagged proteins were derived from the pEF6/MycHis. This allowed us to express each of the transporters cloned either with a N-terminal or a C-terminal 6xHis-tag as well as the untagged form to maximize the chances of expressing a recombinant active transporter. If the 6xHis-tags were not to interfere with the cellular localization or folding of the protein, they would be helpful to quantify their relative expression by western blot analysis. Primers used for PCR amplification and the restriction enzymes used for cloning in the respective plasmids are provided in Supplementary Table 1. When clones were obtained (one single isoform for each of the proteins listed except for human SLC5A10 where two isoforms were cloned—a shorter hSGLT5-iso2; NP_001035915.1 and a longer hSGLT5-iso1; NP_689564.3), the plasmids used to transfect cells and overexpresses the transporter proteins were all checked by sequencing. These plasmids were next transfected in HEK293T cells using JetPEI^®^ as a transfection agent as described previously [25]. To detect expression of the various recombinant proteins, cells were collected after 48 h from 10 cm diameter culture dishes by removing the media, washing with 5 ml PBS, and scraping the culture dish to collect the cells in 0.8 ml/plate of lysis buffer (25 mM HEPES, pH 7.2, leupeptin and antipain 5 µg/ml each and 0.5 mM PMSF). Extracts for Western blot analysis were prepared following two freeze-and-thaw cycles using liquid nitrogen and deoxyribonuclease I (DNAse I) treatment (addition of 10 µl of a solution containing 2 mg/ml DNAse I in 1 M MgSO_4_ per 0.8 ml cell extract) for 30 min on ice. Protein expression of the 6xHis-tagged proteins was visualized by Western blot analysis following protein separation by SDS-PAGE gel (10 µl/well) using mono-clonal anti-His antibody (GE Healthcare) and penta-His antibody (Qiagen) to detect N- and C-terminally 6xHis-tagged SGLT4 and SGLT5, respectively [2].

### Cloning of SGLT4 and SGLT5 in lentiviral vectors and creation of model cell lines overexpressing untagged mouse and human SGLT4 and SGLT5

The coding sequences of mouse and human SGLT4 and SGLT5 that we had already cloned in the pEF6/MycHis expression plasmids described above (untagged proteins) were PCR amplified using the primers provided in Supplementary Table 2. The PCR-amplified DNA fragments contained at their ends DNA sequences recognized by the restriction enzymes XbaI (5’-end) and NotI (3’-end) which we used for cloning in two lentiviral vectors (pUB82 or pUB83 containing the SV40 or CMV promoter, respectively). After sequencing verification, all ten constructed lentiviral vectors (pUB82/83 mSGLT4, mSGLT5, hSGLT4, hSGLT5-iso1 and hSGLT5-iso2) were used to generate recombinant lentiviruses by transient transfection of HEK293T cells with the respective lentiviral vectors and second-generation packaging plasmids psPAX2 and PMD2.G (Addgen #12260 and #12259) using calcium phosphate coprecipitation method [26].

Practically, on day 1, we seeded two 6-well plates (12 wells) each with 8.4 × 10^5^ HEK293T cells, which were transiently transfected on day 2 with the lentiviral vectors (pUB82 or pUB83, either empty or containing our sequences of interest) together with psPAX2 and PMD2.G. The transfection mix contained 3.36 µg of packaging plasmid PSPAX2, 1.7 µg of envelope plasmid PMD2.G, 3.36 µg of transfer expression plasmid DNA and 16.8 µl of 2.5 mM CaCl_2_ all diluted in deionized distilled water to a final volume of 168 µl. This DNA containing solution was next twofold diluted with 2 × HBSS solution under bubbling to create DNA precipitates and added dropwise to each well of HEK293T cells kept at 37 °C in 5% CO_2_. The culture media was changed 6 h after transfection and the recombinant viruses were finally recovered in the cell culture supernatant 24 h later (day 3). At this point, the packaged recombinant lentivirus containing media was recovered, appropriately diluted in cell culture media containing polybrene (4 µg/ml) and subsequently used to transduce the target cells (in our case, HEK293T seeded—0.4 × 10^5^ cells/well—on day 2 on 6-well plates–1 well/condition). After 24 h, on day 4, we began the selection of the transduced HEK293T cells with puromycin (1.5 µg/ml) for 3 more days. Finally, the puromycin containing culture medium was removed and the surviving cells in each well were amplified. Frozen stocks were prepared that could be regularly used in the transport assays.

### Radioactive uptake assays

This assay was used to study (1) the transport of 1,5-AG or mannose by the HEK293T model cell lines that stably overexpressed the various transporters or by the transiently transfected HEK293T cells using JetPEI^®^ as a transfection agent prepared as described above and (2) the substrate specificity of each transporter.

For a typical transporter experiment we proceeded as follows. On day one, stably or 24 h JetPEI^®^ transiently transfected cells were treated with trypsin, resuspended in culture media and counted in order to seed precisely 0.6 × 10^5^ cells/well of a 24 well plate in triplicate for each cell line together with the appropriate control HEK293T cells that did not overexpress the transporters. To minimize cell loss during the various washing steps in the radioactive uptake experiments, the wells where the cells were seeded were previously coated with poly- d-lysine, which improved their adhesion to the plastic surface of the wells. For this, each well of the 24-well plates was treated with 200 µl of a 0.01% poly-l-lysine solution for 10 min, washed 3 times with 1 ml of sterilized water and left to dry for 2 h. Only then could the 24-well plates be seeded with the appropriate cell lines and cultured overnight at 37 °C in 5% CO_2_.

On day two, the radioactive uptake assay could be performed. The cell culture media was removed by aspiration and cells were incubated for 20 min at 37 °C and 5% CO_2_ with a basal medium (10 mM HEPES, pH 7.4, 1 mM CaCl_2_, 1 mM MgCl_2_, 2 mM KCl) containing 140 mM choline chloride to deplete intracellular Na^2+^ ions. This was subsequently replaced with the uptake medium (basal medium + 140 mM NaCl, 25 μM cytochalasin B, 1 mg/ml BSA) containing either radioactive U-[^14^C]-mannose (15,000 cpm/well) or 2-[^3^H]-1,5-AG (30,000 cpm/well) and respectively, non-radioactive mannose or 1,5-AG, at the required concentration in the experiment. Importantly, when measuring the transport of mannose, the uptake media also contained 0.5 mM glucose. This resulted from the need to reduce the background transport of mannose in control HEK293T cells in order to optimize the transport assay for this sugar in our model cell lines. Consequently, after testing various inhibitors (Supplementary Fig. 1), 0.5 mM glucose + 25 µM cytochalasin B appeared as the best combination to decrease efficiently the endogenous transport of mannose in control cells without affecting the transport of mannose in model cell lines overexpressing SGLT4 and SGLT5. Therefore in the radioactive uptake assays for mannose, the uptake medium was: basal uptake medium + 140 mM NaCl, 25 μM cytochalasin B, 0.5 mM glucose and 1 mg/ml BSA to which we added U-[^12^C + ^14^C]-mannose as described above.

Once the uptake medium was added to the cells, the plates were incubated at 37 °C in 5% CO_2_ during the required time. To stop the incubation (after 30 or 60 min during which we could show that the uptake was time dependent for both 1,5-AG and mannose—Supplementary Fig. 2), the radioactive uptake media was carefully removed by aspiration, cells were washed with 1 ml of ice-cold basal media (basal uptake medium with 140 mM NaCl) and lysed in 0.5 ml of 1% Triton-X100 to recover intracellular radioactivity, which we measured in a scintillation counter after mixing with 5 ml scintillation cocktail. In each experiment, the transport activities were calculated after subtracting a “blank” value, corresponding to the radioactivity in the appropriate control cells (HEK293T cells that were stably or transiently transfected with one of the parent plasmids pEF6/MycHisA, pEF6HisB, pUB82 or pUB83 with no inserted cDNA) that were analyzed in parallel.

The transport experiments to study the specificity of SGLT4 or SGLT5, were performed in the presence of 10 µM U-[^14^C + ^12^C]-mannose (for SGLT4) or 2-[^3^H + ^1^H]-1,5-AG (for SGLT5) and increasing concentrations of various sugars tested. The transport experiments to study the inhibition of empagliflozin, dapagliflozin or remogliflozin, on the transport of 1,5-AG by SGLT5 or its variants were performed in the presence of 10 µM 2-[^3^H + ^1^H]-1,5-AG and increasing concentrations of the various gliflozins tested.

### Site directed mutagenesis to create model cell lines overexpressing SGLT5 mutants to study substrate specificity and SGLT5 variants to study 1,5-AG transport activity

SGLT5 mutants were created using the quick-change site directed mutagenesis method that enables effective mutagenesis without subcloning of the amplified PCR products [27]. Complementary primer pairs carrying the desired replacements (Supplementary Table 3) were designed using an online tool (https://www.agilent.com/store/primerDesignProgram.jsp). The PCR protocol was carried out following the manufacturer instructions regarding the KOD HOT Start polymerase kit (TOYOBO™, Novagen). The amplified DNA was cleaned-up from the reactions constituents using the GeneJET PCR purification Kit (Thermo Scientific™) and the size of the DNA fragment were verified by DNA agarose-gel electrophoresis. After eliminating the parental DNA by digesting the purified DNA with the DpnI restriction enzyme, the DNA fragments containing the appropriate changes were transformed in *E. coli* NEB Turbo competent cells (C2984H). Once colonies were obtained, plasmid DNA was finally amplified, purified and sequenced to verify that the required changes had been introduced and eliminate any PCR errors. Next, pUB83-hSGLT5-iso2 as well as the various SGLT5 mutants obtained were used to transduce HEK293T cells using the exact same protocol as described above to create model cell lines overexpressing the different SGLT5 transporters.

## Results

### Construction of model cell lines to investigate the transport properties of SGLT4 and SGLT5 and initial characterization of SGLT4 and SGLT5 by transient transfection in HEK293T cells

**Fig. 1:**
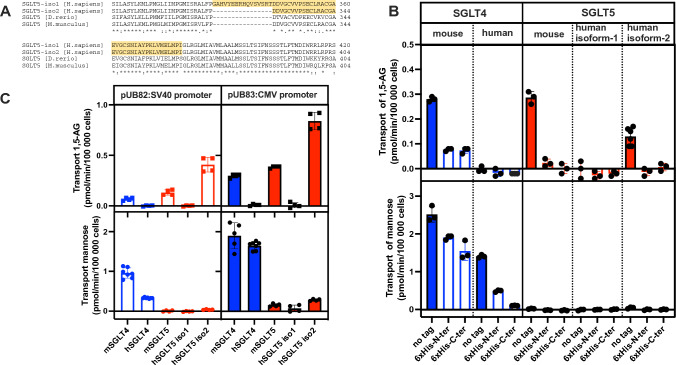
Transport of 1,5-AG and mannose by HEK293T cell overexpressing human and mouse SGLT4 and SGLT5 isoforms. **A** Alignment of human SGLT5/SLC5A10 protein sequences highlighting the 16 amino acid non-conserved N-terminal extension of exon 10 present in SGLT5-iso1. The sequences blasted correspond to: human SGLT5-iso2 (NP_001035915.1) and SGLT5-iso1 (NP_689564.3); mouse SGLT5 (NP_001028399.1); zebrafish SGLT5. **B** Impact of the 6xHis-tag on the transport activity of cells transfected to transiently overexpress SGLT4 or SGLT5. Transport was measured in 24 well plates with 0.6 × 10^6^ cells during 30 min at 37 °C in 5% CO_2_ in the presence of 10 µM 2-[^1^H + ^3^H]-1,5-AG (upper panel) or U-[^12^C + ^14^C]-mannose (lower panel) as described in Materials and Methods. **C** Transport activity for 1,5-AG (upper panel) and mannose (lower panel) of model cell lines overexpressing the untagged transporters in a stable fashion created by lentiviral transduction of HEK293T cells as described in Materials and Methods. Transport activities were measured as in (B) for 60 min. Data corresponds to n = 3 of least two independent experiments. 1,5-AG–1,5-anhydroglucitol

In order to study the specificity of SGLT4 and SGLT5 for 1,5-AG, we cloned the cDNAs that code for both the mouse and the human isoforms. For human SGLT5, among the sequences cloned, we noticed that there are two isoforms: a shorter and more abundant (hSGLT5-iso2; NP_001035915.1) and a longer and less abundant (hSGLT5-iso1; NP_689564.3) that has a non-conserved extension of 16 amino acids at the N-terminal end of exon 10 (Fig. 1A). Of note, it is the longest sequence that is used as the reference sequence in the Genome Aggregation Database—gnomAD. Consequently, and despite the suggestion that hSGLT5-iso2 could be the active isoform, we chose to construct expression plasmids for both human isoforms (hSGLT5-iso1 and hSGLT5-iso2) to test their transport activities. For the other transporters only one isoform was cloned and expressed. Since we were interested in comparing the expression level of the various transporters, we produced fusion proteins containing a 6x-His-tag at the N- or C-terminal end, as well as the corresponding untagged proteins.

Figure 1B illustrates the transport activity of the various proteins on 1,5-AG and mannose measured in transfected cells transiently overexpressing the indicated transporters in the presence of 10 µM substrate and the corresponding radiolabeled substrate. These results indicated that (1) mouse SGLT5 and the human SGLT5-iso2 (the shorter isoform) both transported 1,5-AG; (2) human SGLT5-iso1 (the longer isoform) was not detectably active; (3) the 6xHis-tagged forms of human SGLT5-iso2 were barely active compared to the untagged protein and this despite being expressed as indicated by Western blots (Supplementary Fig. 3). These findings indicated that the addition of the 6xHis-tagged interfered with protein folding, localisation in the membrane, or transport activity.

Transport measurements in SGLT4 expressing cells showed that these transported mannose (and also 1,5-AG, in the case of the mouse transporter). Transport activity was also significantly reduced by the addition of the 6xHis-tags. Moreover, in the cells that transiently overexpressed SGLT5 we could not detect mannose transport (Fig. 1B), while low mannose transport could be detected in stable transfectants overexpressing SGLT5 under the more active CMV promoter (see below; Fig. 1C).

Taken together, we concluded that the study of the transport of sugars by SGLT5 and SGLT4 could only be done in cell lines overexpressing the recombinant untagged forms of the respective transporters. Furthermore, we could also show that only the shorter, conserved human SGLT5 isoform (SGLT5-iso2; NP_001035915.1) was active, the longer and non-conserved isoform (SGLT5-iso1; NP_689564.3) has no transport activity.

### Further characterization of SGLT4 and SGLT5 in stable transfectants

To facilitate transport measurements, we created model cell lines that overexpressed the human transporters in a stable fashion by lentiviral transduction of HEK293T cells. We produced lentiviral particles containing the appropriate plasmids (pUB82 and pUB83) for overexpression of the five untagged transporters under the control of two viral promoters (the less active SV40 present in the pUB82 plasmid and the more active CMV present in the pUB83 plasmid), which we used to create the model cell lines. Transport measurements performed with these cells indicated that transport activities were higher with the CMV promoter driven expression compared with the SV40 promoter driven one (Fig. 1C). Confirming the results obtained in the experiments using cells that transiently overexpressed the transporters, SGLT4 (and even more so the human isoform) was found to better transport mannose than it did 1,5-AG, while the opposite was true for both mouse and human SGLT5.

HEK293T cells transiently or stably overexpressing SGLT4 from mouse (Fig. 1B, C) suggested that unlike the human protein, the mouse transporter was less specific for mannose and also able to transport 1,5-AG. Yet, when the affinity for the transport of 1,5-AG for mouse SGLT4 and SGLT5 was compared, this clearly showed that as for the human transporters, SGLT5 from mouse had over tenfold higher affinity for 1,5-AG than SGLT4 did. Moreover, the Km of mouse SGLT4 was 25-fold lower for mannose than for 1,5-AG and the transport activity of the mouse SGLT4 measured in the presence of 50 µM mannose was barely inhibited by 10 mM 1,5-AG (Supplementary Fig. 4). Together, these results suggest that in mice (as in humans), 1,5-AG is transported by SGLT5 and mannose is mainly transported by SGLT4.

### Specificity and kinetic properties of human SGLT4 and SGLT5.

**Fig. 2:**
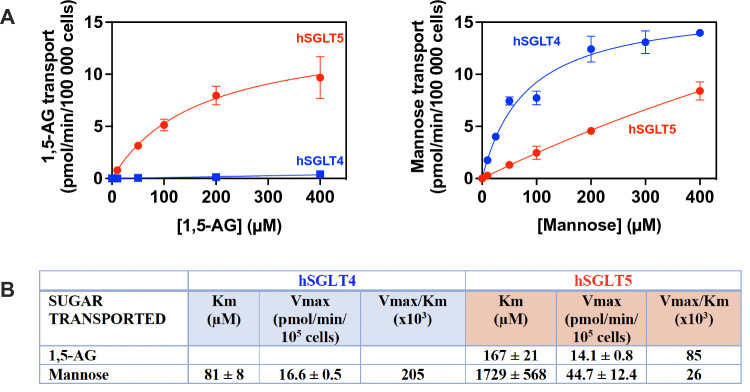
Human SGLT5 transports 1,5-AG while human SGLT4 transports mannose and not 1,5-AG. Saturation curves for the transport of **A** 1,5-anhydroglucitol and mannose by SGLT5 and SGLT4. Transport was measured during 30 and/or 60 min at 37 °C in 5% CO_2_ in 24 well plates with 0.6 × 10^6^ cells overexpressing human SGLT4 or SGLT5 in the presence of shown concentrations of 2-[^1^H + ^3^H]-1,5-AG (left panel) and U-[^12^C + ^14^C]-mannose (right panel). **B** Kinetic constants for both transporters were computed by fitting the data to the Michaelis–Menten model in Prism – GraphPad. The catalytic efficiency is estimated by the Vmax/Km values. Data corresponds to n ≥ 3 in at least 3 independent experiments. 1,5-AG – 1,5-anhydroglucitol

We next focused on the investigation of the kinetic characteristics of the human active isoforms of SGLT4 and SGLT5, because of their possible association with the neutropenias in GSD1b and G6PC3-deficiency. Consequently, we measured the transport of radiolabelled 1,5-AG (2-[^1^H + ^3^H]-1,5-AG) and mannose (U-[^12^C + ^14^C]-mannose) in the HEK293T model cell lines overexpressing human SGLT4 and human SGLT5-iso 2. Figure 2 shows that contrary to what had previously been suggested [18] human SGLT4 does not transport 1,5-AG, but it transports mannose, while SGLT5 best transports 1,5-AG but also, less efficiently, mannose (Fig. 2A). The kinetic properties that were determined (Fig. 2B) indicate that the Km for 1,5-AG (167 ± 21 µM) is in the range of the concentrations measured in blood (± 150 µM; [28]). The Km for mannose was higher resulting in a ratio of Vmax/Km approximately 3.5-fold higher for 1,5-AG than for mannose, confirming that remarkably 1,5-AG was indeed the best of the two substrates for SGLT5.

The kinetic properties of SGLT4 for mannose suggested that this transporter was much better at transporting mannose than was SGLT5, yet due to lack of information on the amount of each transporter that is present at the membrane (and therefore active), the intrinsic transport activity between SGLT4 and SGLT5 cannot be directly compared. If we assume a similar expression of these transporters in our cell lines, we reach the conclusion that SGLT4 is approximately tenfold better than SGLT5 to transport mannose (Fig. 2B).

We also tried to measure fructose transport in SGLT5 overexpressing cell lines, a known substrate for SGLT5 [24, 29]. However, with the technique used, we had a high basal transport of fructose in control cells and only a non-significant increase in fructose transport in cells overexpressing SGLT5. This background entry, together with the low affinity of SGLT5 for fructose (see below) precluded characterization of the transport of radiolabelled fructose using our experimental system. However, fructose transport by SGLT5 is a well-established property of this transporter [24, 29], which is indirectly confirmed by its ability to compete the transport of 1,5-AG (see below).

**Fig. 3:**
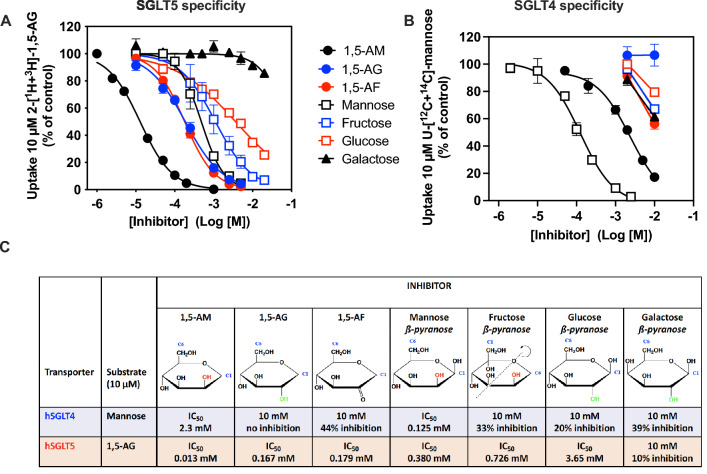
Substrate specificity of human SGLT4 and SGLT5. Inhibition curves of the transport activity of **A** 1,5-anhydroglucitol by human SGLT5 and **B** mannose by human SGLT4. Measurements were performed during 30 and/or 60 min in the presence of 10 µM 2-[^1^H + ^3^H]-1,5-AG or U-[^12^C + ^14^C]-mannose and the indicated concentrations of the inhibitor sugars in 24 well plates with 0.6 × 10^6^ cells overexpressing human SGLT5 or SGLT4, respectively. **C** When shown, the IC_50_ values are derived in Prism – GraphPad from the curves in **A** and **B** and indicate the concentration of the sugars needed to inhibit by 50% the transport activity. When the inhibition was too weak and IC_50_ values could not be derived, the degree of inhibition with 10 mM inhibitor is shown. The structures of the various inhibitory sugars used are shown (Haworth projections) to highlight the absence of the OH group bound to carbon 1 (C1) in 1,5-anhydromannitol, 1,5-anhydroglucitol and 1,5-anhydrofructose, and the orientation of the OH group bound to carbon 2 (left OH shown in red; right OH shown in green). The structure of the molecule of fructose is shown after a rotation along the oxygen 6–carbon 4 axis to indicate its structure similarity with 1,5-anhydromannitol. Data corresponds to *n* = 3 in at least 2 independent experiments. 1,5-AM – 1,5-anhydromannitol; 1,5-AG–1,5-anhydroglucitol; 1,5-AF – 1,5-anhydrofructose

To further characterize the specificity of SGLT5 and SGLT4, we investigated the competition exerted by several unlabelled sugars and polyols on the uptake of radiolabelled 1,5-AG by SGLT5 (Fig. 3A) and radiolabelled mannose by SGLT4 (Fig. 3B). Remarkably, 1,5-anhydromannitol was the most potent competitor for SGLT5 (Ki = 13 µM), followed by 1,5-AG, 1,5-anhydrofructose, mannose, fructose, glucose and galactose. The preference for 1,5-anhydromannitol over 1,5-AG (12-fold difference in Ki) and for mannose over glucose (tenfold difference in Ki) indicated that in the Haworth projection, the left orientation of the OH group bound to C2 is preferred by SGLT5. Similarly, the preference of 1,5-anhydromannitol over mannose (25-fold difference in Ki) and of 1,5-AG over glucose (20-fold difference in Ki) indicates that SGLT5 prefers to transport compounds that do not have an OH group on C1 (Fig. 3C).

Of note, fructose in its *β*-pyranose form (which represents ≈ 70% of the fructose in solution) shows a similar structural arrangement of the OH groups on C1 and C2 as in 1,5-anhydromannitol. This can be easily seen if the fructose structure is flipped around its O6–C4 axis: there is no OH group bound to C6 (equivalent to C1 in 1,5-anhydromannitol) and the OH group bound to C5 has the same spatial orientation as the OH bound to C2 of 1,5-anhydromannitol. This may explain the relatively high affinity of SGLT5 for fructose, compared to glucose, which has an OH group bound to C1 and an OH group bound to C2 in the right orientation (see Fig. 3C). 1,5-AG is an intermediary case with no OH group in C1 (as appearing to be preferred by SGLT5) but an OH group in the less preferred orientation (the left one) bound to C2. The lower affinity of SGLT5 for galactose compared to glucose indicates that the orientation of the OH group on C4 is also important.

A similar specificity study on SGLT4 did not allow us to identify a better ligand than mannose (Fig. 3B). Significant competition was observed with 1,5-anhydromannitol, though not with 1,5-AG, indicating that SGLT4 (as SGLT5) also prefers that the OH group bound to C2 is in the left orientation. The lower affinity for 1,5-anhydromannitol than for mannose indicates that, contrary to SGLT5, the presence of an OH group bound to C1 (as in mannose) favours binding in SGLT4 (Fig. 3C).

**Fig. 4:**
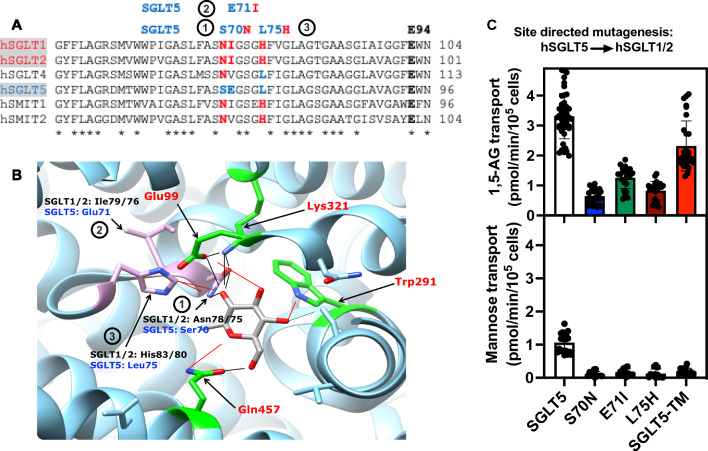
Site directed mutagenesis of specific features of SGLT5 binding pocket. **A** Multiple sequence alignment of selected human SGLT5 homologs showing strictly conserved residues in the binding pocket that possibly interact with the OH groups carried by carbons 1 and 2 of the hexose substrate molecules. The indicated residues in SGLT5 (1-Ser70, 2-Glu71 and 3-Leu75) were replaced individually or all together (as shown in panel C) by the corresponding residues in SGLT1 and SGLT2 (1-Asn78/75, 2-Ile79/76 and 3-His83/80). The protein sequence of the homologous proteins were aligned using Clustal Omega (https://www.ebi.ac.uk/Tools/msa/clustalo/). **B** SGLT2-MAP17 cryo-EM structure (code PDB: 7vsi [33]) of the substrate binding pocket crystalized with empagliflozin in it (for clarity we hid the aglycone part of the empagliflozin molecule which is normally linked to carbon 1), showing the position of 1-Asn75, 2-Ile 76 and 3-His80 and depicting their possible interactions (black lines: interactions shown in the structure proposed by Niu et al. [33]; red lines: interactions suggested in Sala-Rabanal et al*.* [30]) with the substrate and other residues around the binding pocket. **C** Impact of replacing the indicated residues in SGLT5 by the equivalent ones in SGLT1 and SGLT2 on the transport activities of 1,5-AG and mannose. SGLT5-TM corresponds to a mutant SGLT5 carrying all three mutations (S70N–E71I–L75H). Transport activities were measured in the presence of 50 µM 2-[^1^H + ^3^H]-1,5-AG or U-[^12^C + ^14^C]-mannose. Data corresponds to *n* = 3 in at least 3 independent experiments. 1,5-AG–1,5-anhydroglucitol

Taken together our data indicates that both SGLT4 and SGLT5 were selected to transport a sugar/polyol (mannose/1,5-anhydromannitol) with an OH group bound to C2 that has a left orientation (contrary to SGLT1 and SGLT2). This is possibly due to the replacing of His83/His80 (respectively in SGLT1/2) by a leucine (as in SGLT4/5) which might invert the specificity with respect to the binding of the OH group on C2. It is possible that in SGLT4 (and maybe also in SGLT5) the substrate-binding glutamate corresponding to Glu99 in SGLT2 (Fig. 4B and Supplementary Fig. 5) is well oriented to do this. In addition, SGLT4 prefers substrates with an OH group on C1 while the opposite is true for SGLT5.

### Site directed mutagenesis in SGLT5 ascribes the specificity of SGLT5 for 1,5-AG to some specific residues

The establishment of the structure of human SGLT1 [30–32] and SGLT2 [33] has allowed the identification of the residues in the glucose binding pocket in these two transporters. A multiple alignment (Supplementary Fig. 5) shows that, as expected, the residues that bind the OH groups carried by C3, C4, and C6 of the hexose are strictly conserved among all the human proteins of this family including SGLT4 and SGLT5, but that this is not the case for residues that potentially interact with the OH groups bound to C1 and C2 of the hexose (Fig. 4A, B). Thus, in SGLT1/2, Asn78/75 which binds the OH group on C2 in glucose (Fig. 4B) is replaced by a serine in SGLT5 (S70), while the neighbouring isoleucine (Ile79/76) in SGLT1/2 is replaced by a glutamate (Glu71) (Fig. 4A), which is strictly conserved among the SGLT5 orthologs (Supplementary Fig. 6). Furthermore, His80/83 in SGLT1/2, which is in the vicinity of the first two residues and could also bind the OH group on C2 in SGLT1 [30] (Fig. 4B), is replaced by a leucine in both SGLT4 (Leu92) and SGLT5 (Leu75) (Fig. 4A, B). This suggested that the difference in specificity could be potentially ascribed to these residues.

To test this possibility, we restored by site-directed mutagenesis in the SGLT5 sequence the three residues found in SGLT1 and SGLT2 (S70N, E71I, L75H) and compared the transport activity and the specificity of the resulting proteins to those of wild-type SGLT5. Changing any of these three residues, decreased the ability of SGLT5 to transport the two substrates 1,5-AG and mannose (Fig. 4C). Surprisingly, when all three residues were changed together, the triple mutant (SGLT5-TM) recovered some of its ability to transport 1,5-AG, yet less efficiently than wild-type SGLT5 (Fig. 4C). In agreement, competition experiments indicated that SGLT5-TM had lost 250-fold affinity for 1,5-anhydromannitol, tenfold affinity for mannose but also fructose and it had gained 30-fold affinity for glucose and galactose, while still being inhibited by 1,5-AG (Supplementary Fig. 7). Altogether, this indicated that SGLT5-TM had acquired transport properties that resemble, though are not identical to, those of SGLT1/2, indicating that in SGLT1 and SGLT2 residues other than those that we have mutated also contribute to substrate recognition.

Examining the effect of the individual mutations did not allow us to derive a simple picture about the relative importance of the three residues for binding the different sugars and polyol, but it showed that the presence of Ser70, Glu71 and Leu75 is certainly very important for the ability of SGLT5 to transport 1,5-AG, mannose, and fructose as well as for its remarkable affinity for 1,5-anhydromannitol (Supplementary Fig. 7).

### SGLT5 variants associated with lower 1,5-AG in blood decrease the ability of SGLT5 to transport 1,5-AG

**Fig. 5:**
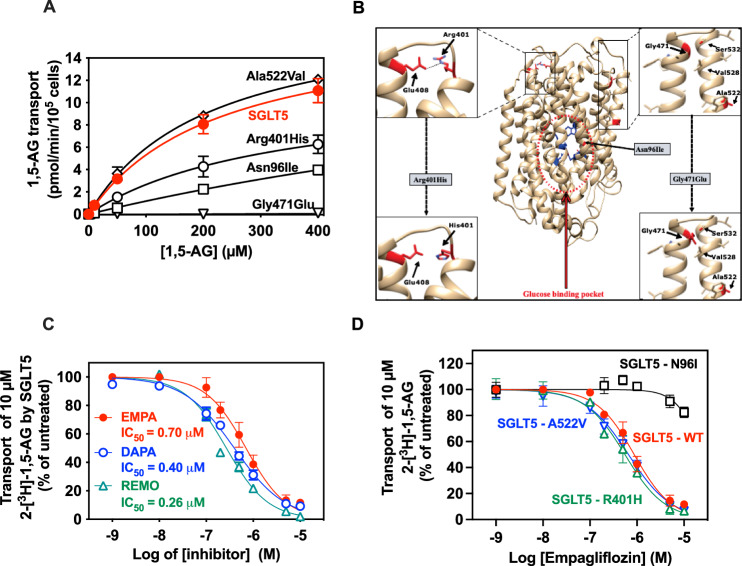
Impact of mutations in SGLT5 on the transport activity for 1,5-AG and on the affinity for gliflozins. **A** Saturation curves for the transport of 1,5-AG by various model cell lines overexpressing the indicated SGLT5 mutants (see Table 1). 1,5-AG transport was measured in the presence of the indicated concentrations of 2-[^1^H + ^3^H]-1,5-AG during 30 and/or 60 min. Data corresponds to n = 3 in at least 2 independent experiments. **B** Localisation and illustration of the possible impact of the mutations present in the variants of human SGLT5-iso2 (NP_001035915.1) modelled using AlphaFold [34, 35]. **C** Inhibition curves of the transport activity of 1,5-AG by human SGLT5 measured in the presence of 10 µM 2-[^1^H + ^3^H]-1,5-AG and increasing concentrations of the indicated gliflozins. The IC_50_ values are derived in Prism – GraphPad from the inhibition plots shown and indicate the concentration needed of each gliflozin to inhibit by 50% the transport activity. **D** Impact of mutations found in SGLT5 variants on the ability of empagliflozin to inhibit the transport of 1,5-AG. Data corresponds to *n* = 3 in at least 2 independent experiments. EMPA - empagliflozin, DAPA - dapagliflozin, REMO - remogliflozin and 1,5-AG–1,5-anhydroglucitol

Mutations of SGLT5 have been associated with lower levels of 1,5-AG in the general population (Asn96Ile; Gly471Glu) [21–23] and in a patient with G6PC3 deficiency (Arg401His) [4]. Since none of these studies addressed the functional impact of these mutations in the transport of 1,5-AG by SGLT5, we produced the relevant mutant forms of SGLT5 by site directed mutagenesis and created the appropriate model cell lines to measure 1,5-AG transport. As a control, we chose to test the effect of a frequent SNP (Ala522Val; rs12604020) found in SGLT5 (which is shown as Ala538Val in the gnomAD database). The allele frequency of the Ala522Val variant is 0.068 (in the total population; 0.020 in the European population; Supplementary Table 4) and, unlike the previous SNPs, it has not been associated with changes in the concentration of 1,5-AG in blood. Three of the mutations associated with lower 1,5-AG in blood decreased (Asn96Ile, Arg401His) or abolished (Gly471Glu) the ability of SGLT5 to transport 1,5-AG, while no effect was observed for the Ala522Val replacement (Fig. 5A). The mutants for which we could still measure the transport of 1,5-AG, showed a transport efficiency that was reduced by twofold (Arg401His) or sixfold (Asn96Ile) when compared to SGLT5, while this was not the case for the control mutant (Ala522Val) (Fig. 5A, and Table 1).

**Table 1:** Kinetic properties of the transport of 1,5-anhydroglucitol by the SGLT5 variants associated with reduced levels of 1,5-anhydroglucitol in blood

Transporter (variant ID)^a^	Allele frequency (GnomAD)	Associated with [1,5-AG] in blood	Km for 1,5-AG (µM)	*V*_max_ (pmol/min/10^5^cells)	*V*_max_/Km (× 10^3^)
hSGLT5 WT (NP_689564.3)	_	_	226 ± 7	17.3 ± 0.3	76.5
Asn96Ile (rs148178887)	0.27% (746/280762)	Yes [23]	1565 ± 302	19.5 ± 3	12.4
Arg401His (rs754390288)	0.0024% (6/249474)	Yes [4]	331 ± 13	11.4 ± 0.2	34.4
Gly471Glu (rs61741107)	0.46% (1260/272154)	Yes [23]	No transport	No transport	No transport
Ala522Val (rs12604020)	6.8% (19021/279874)	No	210 ± 12	18.3 ± 0.5	87

^a^The variant IDs can be identified in NCBI in the data base of human genomic Structural Variation (dbVar)

Figure 5B illustrates the impact of the amino acid changes in the AlphaFold [34, 35] predicted structure of human SGLT5-iso2. The highly conserved residue Asn96, for which we noted a clear increase in the Km for 1,5-AG when it was replaced by an isoleucine, is in the glucose binding pocket and very close to the conserved 1,5-AG/glucose binding residue Glu94 (SGLT5)/Glu99 (SGLT2)/Glu102 (SGLT1) [30, 31] (see sequence alignment in Supplementary Fig. 5). The Arg401His mutation replaces a highly conserved arginine (*α*-helix 8) that forms a salt bridge with the also strictly conserved Glu408 in the neighbouring *α*-helix 9. This salt bridge presumably serves to maintain the cohesion between the two *α*-helixes, and it is likely that its replacement by a shorter and less protonated histidine weakens this interaction (Fig. 5B). The Gly471Glu mutation in SGLT5-iso2 replaces a highly conserved glycine (Supplementary Figs. 5 and 6) present in *α*-helix 11 which is expected to cause a side-chain clash with the also highly conserved Ser532 and possibly also Val528 in the facing *α*-helix 12 in SGLT5 (Fig. 5B). In any case, the presence of a glutamate side chain in the *α*-helix 11 would profoundly perturb the hydrophobic interactions that maintains together the two α-helixes, which is in agreement with the failure of the Gly471Glu mutant to transport 1,5-AG.

Whether the effect of these mutations is to reduce the expression, the stability or the intrinsic activity of SGLT5 is unknown and difficult to investigate in the absence of specific antibodies against SGLT5. Yet, the fact that all mutations that are accompanied by a reduction of 1,5-AG concentrations in humans, also cause a decrease in SGLT5 transport activity in our in vitro assays, indicate that they have a similar effect in vivo, whatever the mechanism of this effect is.

### Gliflozins directly inhibit the transport of 1,5-AG by SGLT5

It is generally admitted that the effect of gliflozins on 1,5-AG excretion is indirect and caused by the enhanced glucosuria that results from the inhibition of the renal transporter of glucose, SGLT2, in the proximal tubule. The increase in glucose, in turn inhibits competitively the transport of 1,5-AG by the 1,5-AG transporter [1, 7, 36, 37]. Gliflozins that are used to inhibit SGLT2 and treat diabetes are very specific, but not totally specific for the SGLT2 transporter, they also act on other members of the SGLT family with lower affinity [24, 38]. To better appreciate the possibility that part of the effect of gliflozins on 1,5-AG excretion in vivo might also be due to a direct effect on SGLT5, we tested this with the pathophysiologically relevant substrate, 1,5-AG.

We tested the inhibition of SGLT5-mediated 1,5-AG transport in the presence of 10 µM 2-[^1^H + ^3^H]-1,5-AG with three gliflozins. Two of these (empagliflozin and dapagliflozin) are currently already repurposed in the treatment of the neutropenias present in GSD1b [3, 4, 14–17] and G6PC3-deficiency [4] and the third one (remogliflozin) was shown as being less specific for SGLT2 [24, 38]. From tests performed with a series of gliflozin concentrations, we noted that the most potent inhibitor was remogliflozin (IC_50_ = 0.27 µM) followed by dapagliflozin (IC_50_ = 0.40 µM) and empagliflozin (IC_50_ = 0.70 µM) (Fig. 5C), which is in good agreement with the data obtained by Grempler and co-workers [24, 38]. Of note, using the equation for competitive inhibition, we calculate that the IC_50_ values that we have obtained at 10 µM 1,5-AG would be about twice higher if we had measured them at a concentration of 1,5-AG corresponding to the Km. As we found, but using mannose as a substrate, Grempler and co-workers [24, 38] confirm that the selectivity of remogliflozin is the lowest, that it is intermediate with dapagliflozin and that it is the highest with empagliflozin. Hence, remogliflozin may be the best gliflozin to produce a direct effect on 1,5-AG excretion (as is required to treat the neutropenias in GSD1b and G6PC3 deficiency), depending of course on its effective concentration in the urinary filtrate. Accordingly, it is also very relevant that the IC_50_ values obtained are in the range of the concentration of empagliflozin and remogliflozin reported in plasma of patients taking these drugs [39, 40].

We have also examined if the mutations found in the SGLT5 variants (Fig. 5A and Table 1) affected the affinity for empagliflozin, since this could also have an impact in the response to treatment in the case a patient carried one of these variants. As shown in Fig. 5D, the mutations Ala522Val and Arg401His did not affect significantly the concentration of empagliflozin needed to inhibit SGLT5, while the mutation Asn96Ile considerably decreased (by more than tenfold) the inhibition exerted by empagliflozin. This is not surprising, since Asn96 in SGLT5 is a highly conserved residue among the SGLT family members and, in SGLT2, the corresponding residue (Asn101), is in the proximity of the glucose binding site (see Fig. 5B and [31–33]. Consequently, when examining the structure of human SGLT2 [P31639 (SC5A2_HUMAN)] in AlphaFold [34, 35], we noticed that the side-chain of Asn101 in SLGT2 appears to interact with the side chain of Thr283 in a neighbouring helix. This could help to maintain the structure of the glucose binding site, particularly the position and orientation of Glu99 (see Fig. 4B), which binds the OH group bound in C3 of the sugar substrate in the whole SGLT family (Supplementary Fig. 5). By contrast, the other mutated residues in SGLT5 are far away from the empagliflozin binding site (Fig. 5B), in support to the observation that their replacement does not affect empagliflozin binding.

## Discussion

### The renal 1,5-AG transporter is SGLT5/*SLC5A10* and not SGLT4/*SLC5A9*

Like other members of the SGLT family, SGLT5 is present in the apical membrane of the kidney tubule epithelium [41] while the identity of the transporter(s) allowing 1,5-AG to cross the basolateral membrane is still unknown. Its ability to transport mannose and fructose has been described in several studies [24, 29], but its role in the transport of 1,5-AG has been neglected in the specificity studies made for this transporter. This role was suggested, however, by the link between SNPs or mutations found in the *SLC5A10* gene (coding for the SGLT5 transporter) that were shown to be genetically associated with the level of 1,5-AG in blood [21–23]. Furthermore, in vivo studies by Yamanouchi et al*.* [42], identified in rat renal tubule a common reabsorption system for 1,5-AG, fructose and mannose that is distinct from the major glucose reabsorption system. In the light of our results this reabsorption system is most likely mediated by SGLT5, particularly since the reabsorption of 1,5-AG alone was little influenced by the presence of larger amounts of fructose or mannose, whereas the reabsorption of fructose or mannose was markedly inhibited by the presence of 1,5-AG [42].

In agreement, the present study shows that SGLT5 is an excellent 1,5-AG transporter. Remarkably, SGLT5 kinetic properties indicated that 1,5-AG is better transported than are mannose (or fructose), by a factor of 3–4 if the Vmax/Km ratio is used as a criterion. Of note, we were unable to determine accurately the Km and *V*_max_ for the transport of fructose due to the already high transport activity for fructose that was present at baseline in the HEK293T control cell line. Unlike for mannose and 1,5-AG, the baseline transport of fructose was not inhibited with cytochalasin B and/or glucose (Supplementary Fig. 1). Yet, we could deduce the affinity of SGLT5 for fructose (and compare it to mannose, 1,5-AG and to other hexoses) from the competition experiments performed (Fig. 3C). Consequently, we could show that among the hexose molecules tested, 1,5-anhydromannitol, the 1,5-anhydropolyol that has the same orientation of the OH group on C2 as in mannose (but has no physiological relevance), is by far the best substrate for SGLT5. This suggests that SGLT5 prefers to transport hexoses with no OH group on C1 and with an OH group on C2 in the same orientation as in mannose.

Similar studies performed on SGLT4 (Fig. 3C) indicate that its preferred substrate is by far mannose, and that 1,5-AG and 1,5-anhydromannitol are much poorer substrates. This indicates that SGLT4 has a real preference for compounds that carry an OH group on C2 with the same orientation as in mannose but that unlike for SGLT5, these compounds should also carry an OH on C1.

The difference in specificity of SGLT5 compared to SGLT1/2 could be ascribed by site directed mutagenesis to three residues that are predicted to participate in the binding of the C1-C2 hexose/polyol substrate, based on structural models of SGLT1 [30–32] and SGLT2 [33]. Thus, the replacement of Ser75, Glu76 and Leu80 (in SGLT5) by Asn, Ile or His (as in SGLT1/2) led to restoration of an SGLT5 (SGLT5-TM) with high affinity for glucose, as observed with SGLT1 and SGLT2, and a low affinity for 1,5-anhydromannitol (Supplementary Fig. 7).

SGLT4 was previously shown to transport 1,5-AG, in addition to mannose, fructose and galactose, but the studies were performed using concentrations of 1,5-AG (30 mM) [18] that are far above the physiological concentration of this substrate (± 150 µM) [7]. In our hands, the transport of 1,5-AG by SGLT4 was undetectable when tested at concentrations in the physiological range, while the transport of mannose could be easily measured. Regarding the transport specificity of SGLT4, our data are in good agreement with the previous report of Tazawa et al. [18], in which transport was measured with a radiolabelled glucose analogue (*α*-methyl-d-glucopyranoside) and the competition with various sugars showed the following order of affinity: d-mannose >  > d-glucose > d-fructose = 1,5-AG > d-galactose. As SLC5A9 (the gene coding for SGLT4) is highly expressed in the intestine and only barely detected in the renal proximal tubule, its main physiological function is likely related to the intestinal absorption of mannose from the food. To date there are no SGLT4 deficiencies reported and no SGLT4 knockout mouse models, which precludes us from commenting on its physiological importance.

We have not investigated in the present manuscript the possibility that MAP17, a protein known to interact with SGLT2 and to increase its transport activity [43] might also affect the activity of SGLT5. This interaction could explain an earlier finding that MAP17 stimulates a Na^+^-dependent transport of mannose catalyzed by an unidentified transporter, that was suggested to be different from SGLT1, SGLT2 and SGLT3 [44]. Retrospectively, this might well be SGLT5, which is highly expressed in rat kidney. Even if physiologically SGLT5 interacted with MAP17, we do not believe that this will affect its specificity, since in our cell models, this specificity is very similar to that deduced from the studies performed in vivo in rats [37, 42].

### In vivo*,* SGLT5 is the main renal transporter of 1,5-AG

The finding that low levels of 1,5-AG in the human population correlate with the presence of variants in SLC5A10, the gene coding for SGLT5 [21–23] strongly argues to support our conclusion that SGLT5 is the physiological 1,5-AG transporter in the kidney. We now show that the mutations found to be associated with low levels of 1,5-AG in blood (see Table 1), decrease the transport activity for 1,5-AG of our model cell lines that overexpress these SGLT5 mutants. It is remarkable that heterozygous subjects for the mutant SGLT5 (with only one wild type allele) show a decrease in the blood concentration of 1,5-AG close to 50%. This suggests that in the proximal tubule, the capacity of 1,5-AG reuptake by SGLT5 is not in large excess.

A previously reported finding [37] that 1,5-AG excretion can be enhanced in rats by parenteral administration of 1,5-anhydromannitol also supports the idea that SGLT5 is the main 1,5-AG transporter in the kidney. If present in the urinary filtrate, 1,5-anhydromannitol is indeed expected to compete with 1,5-AG for the transport carried out by SGLT5, and therefore induce excretion of 1,5-AG. This should certainly not be the case, if the renal reabsorption of 1,5-AG was being carried out by SGLT4, which has 200-fold less affinity for 1,5-anhydromannitol compared to SGLT5 (Fig. 3C).

Our finding that 1,5-AG is very well transported by SGLT5 provides an answer to the long-standing observation that 1,5-AG is efficiently recaptured in the kidney, and remains in the organism during weeks. The finding that high glucose acts as a competitor for 1,5-AG (Fig. 3C) accounts for the observation that 1,5-AG is eliminated in urine in uncontrolled diabetes [10–12]. It also accounts for the fact that inhibitors of SGLT2 (that increase the urinary excretion of 1,5-AG) also lower blood 1,5-AG [45]. Accordingly, SGLT5 is expressed in the S2-S3 segment of the proximal tubule, i.e. downstream of SGLT2, which is expressed in the S1 segment [46]. Gliflozins cause therefore a marked increase in the concentration of glucose to which SGLT5 is exposed, explaining its indirect inhibition by SGLT2-inhibitors. However, since gliflozins also exert some inhibition of the transport of 1,5-AG by SGLT5 (Fig. 6A and [38]), one must also consider that physiologically, part of the enhancement in the excretion of 1,5-AG could be due to a direct inhibition of SGLT5 by gliflozins themselves.

This aspect of the direct inhibition of SGLT5 by gliflozins can be relevant when one considers the repurposing of gliflozins to treat the neutropenia caused by deficiencies in G6PC3 and G6PT [3, 4]. In order to decrease the toxicity of 1,5-AG6P that accumulates in neutrophils of these patients, which is due to its inability to be dephosphorylated, many patients are now treated with gliflozins to decrease the concentration of 1,5-AG in blood [3, 4, 14–17, 47], which is the precursor of 1,5-AG6P in neutrophils. Our studies indicate therefore that the urinary excretion of 1,5-AG is mainly secondary to the hyperexcretion of glucose by the kidneys, but possibly also to a direct inhibition of SGLT5 by the gliflozins used to treat the patients. As such, the presence of a direct effect might justify raising the dose of SGLT2-inhibitors in patients whose 1,5-AG level is particularly elevated, but this has to be balanced with the risk of inducing any possible side-effects and toxicity. Furthermore, our results also hint that the less specific SGLT2-inhibitor remogliflozin [48], versus the commonly used empagliflozin, could be a better gliflozin to be repurposed to treat neutropenia in G6PC3-deficient and GSD1b patients. It could therefore be relevant to document the impact of remogliflozin on decreasing 1,5-AG in blood in comparison with the more commonly used empagliflozin and dapagliflozin. If 1,5-AG levels were to be lower, this suggest that indeed remogliflozin could be a better gliflozin to prescribe for the SGLT2-therapy that is now used to treat neutropenia in GSD1b and G6PC3-deficient patients [3, 4, 14–17, 47].

As a direct consequence of this work, it appears that the best option to improve the elimination of 1,5-AG would be to develop and use a specific SGLT5-inhibitor. In this respect, phlorizin-like molecules in which the OH group on C2 of the sugar moiety is in the l-orientation (rather than in the d-orientation) could be lead compounds for such a class of inhibitor. Based on the difference in affinity observed with hexoses (glucose vs mannose) and polyols (1,5-AG vs 1,5-anhydromannitol), it is anticipated that derivatives of phlorizin carrying a glycoside moiety with a mannose-/1,5-anhydromannitol-like structure would have at least a tenfold higher affinity for SGLT5 than those with the classical glucose-like glycoside moiety (such as the available SGLT2-inhibitors). Optimizing the aglycone moiety might eventually lead to a potent SGLT5-inhibitor.

### Does the efficient reuptake of 1,5-AG by SGLT5 imply that this compound has a physiological function?

It is remarkable that the most efficient physiological substrate of SGLT5 is 1,5-AG. This could indicate that natural selection found an advantage in avoiding elimination of 1,5-AG, which raises the question of whether 1,5-AG might have an (unknown) physiological function.

Yet, several arguments plead against this possibility. Firstly, the occurrence of 1,5-AG in vertebrates has been known for more than 30 years, and no physiological function of 1,5-AG has ever been described. In addition, there is virtually no metabolism of 1,5-AG: it is slowly phosphorylated by side activities of hexokinases and/or ADPGK and readily dephosphorylated by an efficient system in the endoplasmic reticulum that comprises the G6PT and the phosphatase G6PC3 [2, 5, 49]. No other metabolism is known in mammals [50]. Moreover, no clinical symptoms linked to deficiency of 1,5-AG have ever been described despite the fact that millions of diabetic patients worldwide have been treated with gliflozins.

In view of these observations, it appears likely that the selection of SGLT5 as a useful transporter in the kidney is based on its ability to transport efficiently both mannose and fructose, two of the most important sugars (besides glucose and galactose) that are present in food, absorbed in the intestine and therefore filtrated in the kidney. In this respect, the urinary excretion of fructose was shown to be a major feature of an SGLT5-knockout mouse model [29], suggesting that a physiological function of SGLT5 could be to prevent the presence of fructose in urine. This likely contributes to limit bacterial growth in the urinary track and to decrease urinary infections. One other possible role for SGLT5, could be to limit the urinary loss of mannose, which plays an important role in protein glycosylation [51]. Unfortunately, mannose levels were not measured neither in urine nor in blood in the SGLT5-knockout mouse model [29].

It is tempting to consider that it is the structural constraints to achieve fructose and mannose transport (OH orientation on C2 as in mannose; lack of OH on C1 as in the *β*-pyranose conformation of fructose—see Fig. 3C) that most likely explain that SGLT5 best transports 1,5-anhydromannitol (which is physiologically absent) and slightly less well 1,5-AG (which is physiologically present). Consequently, it appears that “the price to pay” for having a renal transporter for fructose (and maybe also mannose), like SGLT5, is that one also keeps 1,5-AG in the body, which is not a problem provided there is no deficiency in G6PT or G6PC3. In addition, the fact that the very best substrate of SGLT5 is a non-physiological molecule (1,5-anhydromannitol) supports the idea that an apparent efficient selection does not necessarily imply that a best substrate is a physiologically important molecule.

### Mutations in SGLT5 are relatively frequent

The presence of mutations in SGLT5 that affect the 1,5-AG concentration in blood [4, 21–23] seem to be quite common in the population. If we just take into account the mutations that have been tested in the present work, their allele frequency amounts to 0.00266 (Asn96Ile), 0.00463 (Gly471Glu) and 2.41e−5 (Arg401His) (Table 1 and Supplementary Table 4). If we now add the mutations causing a premature stop codon that are described in the gnomAD database (total allele frequency of 0.00240), we come to the conclusion that at least 2% of the population has a mutation that decreases the concentration of 1,5-AG in blood by about 50%. This does not take into account the many missense mutations also listed in the gnomAD database (Supplementary Table 4), some of which certainly result in the inactivation of SGLT5.

This high frequency of mutations will have important implications for the treatment of neutropenias linked to G6PC3 and G6PT deficiencies, as a non-negligible fraction of the patients may have a lower blood level of 1,5-AG than others. In their case, they likely have a milder form of neutropenia that can be efficiently treated with a lower dose of SGLT2-inhibitors. This was indeed the case for a recently reported G6PC3-deficient patient who carries the heterozygous mutation Arg401His in SLC5A10, the gene coding for SGLT5 [4]. In the light of these results, SGLT5 can therefore to be considered a modifier gene for the neutropenia in G6PC3-deficient and GSD1b patients, which certainly underlines the importance of measuring blood 1,5-AG before and in the follow-up of the treatment of the neutropenia in these patients.

The frequency of the mutations that lower 1,5-AG in blood also implies that the use of 1,5-AG as a way of monitoring a good glycaemic control in diabetic patients [8, 9] has to be taken with caution. We advise SGLT5 sequencing in the patients that show a discrepancy between good glycaemic control (as assessed by repeated blood glucose measurement or HbA1c or serum fructosamine measurements) and an abnormally low 1,5-AG level.

In conclusion, SGLT5 is the main 1,5-AG transporter in kidney. Consequently, the fairly frequent inactivating mutations in SGLT5, will not only favourably impact the neutropenia associated with G6PC3 and G6PT deficiency, but they also imply, that using the blood concentration of 1,5-AG as a marker to test for glucose excursions in the diabetic population cannot be trusted in individuals harbouring SGLT5 inactivating mutations. Finally, phlorizin derivatives with mannose-like (instead of a glucose-like) glycoside moiety are likely to be powerful agents to lower the 1,5-AG concentration in blood.

### Supplementary Information

Below is the link to the electronic supplementary material.

Supplementary file1 (PDF 2914 KB)

## Data Availability

The data presented in this study are available on the request from the authors

## Abbreviations

1,5-AG: 1,5-Anhydroglucitol
GSD1b: Glycogen storage disease type 1b
1,5-AG6P: 1,5-Anhydroglucitol-6-phosphate
ADPGK: ADP glucokinase
G6PT: Glucose-6-phosphate transporter
G6PC3: Glucose-6-phosphatase catalytic subunit 3
SGLT2: Sodium-glucose cotransporter-2
SGLT4: Sodium-glucose cotransporter-4 (encoded by the gene *SLC5A9*)
SGLT5: Sodium-glucose cotransporter-5 (encoded by the gene *SLC5A10*)
HEPES: 4-(2-Hydroxyethyl)-1-piperazine ethanesulfonic acid
PMSF: Phenylmethylsulfonyl fluoride
DNAse I: Deoxyribonuclease I
HEK293T: Human embryonic kidney 293 cells with a mutant SV40 large T antigen
gnomAD: Genome aggregation database
SNP: Single cell polymorphism
SNC4: Severe congenital neutropenia type 4
