# Supplementary material for: SGLT5 is the renal transporter for 1,5-anhydroglucitol, a major player in two rare forms of neutropenia
Source: Cell Mol Life Sci. 2023 Aug 18;80(9):259. doi: 10.1007/s00018-023-04884-8 (PMC10439028; doi:10.1007/s00018-023-04884-8)
Supplement: Supplementary file 1 — Supplementary file1 (PDF 2914 KB) [file 18_2023_4884_MOESM1_ESM.pdf]

Supplementary Information for:

SGLT5 is the renal transporter for 1,5-anhydroglucitol, a major player in two rare forms of neutropenia

Jennifer Diederich[1], Pierre Mounkoro[1], Hernan A. Tirado[1], Nathalie Chevalier[1], Emile Van Schaftingen[1] and Maria Veiga-da-Cunha[1*]

[1]Metabolic Research Group, de Duve Institute and UCLouvain, B-1200 Brussels, Belgium.

Correspondance

*Maria Veiga-da-Cunha, de Duve Institute, 75, Av. Hippocrate, 1200 Brussels, Belgium;
email: maria.veiga@uclouvain.be
Tel : +327647559

[1]Metabolic Research Group, de Duve Institute and UCLouvain, B-1200 Brussels, Belgium.

Supplementary Figures : 1 - 7

Supplementary Tables :1 – 4

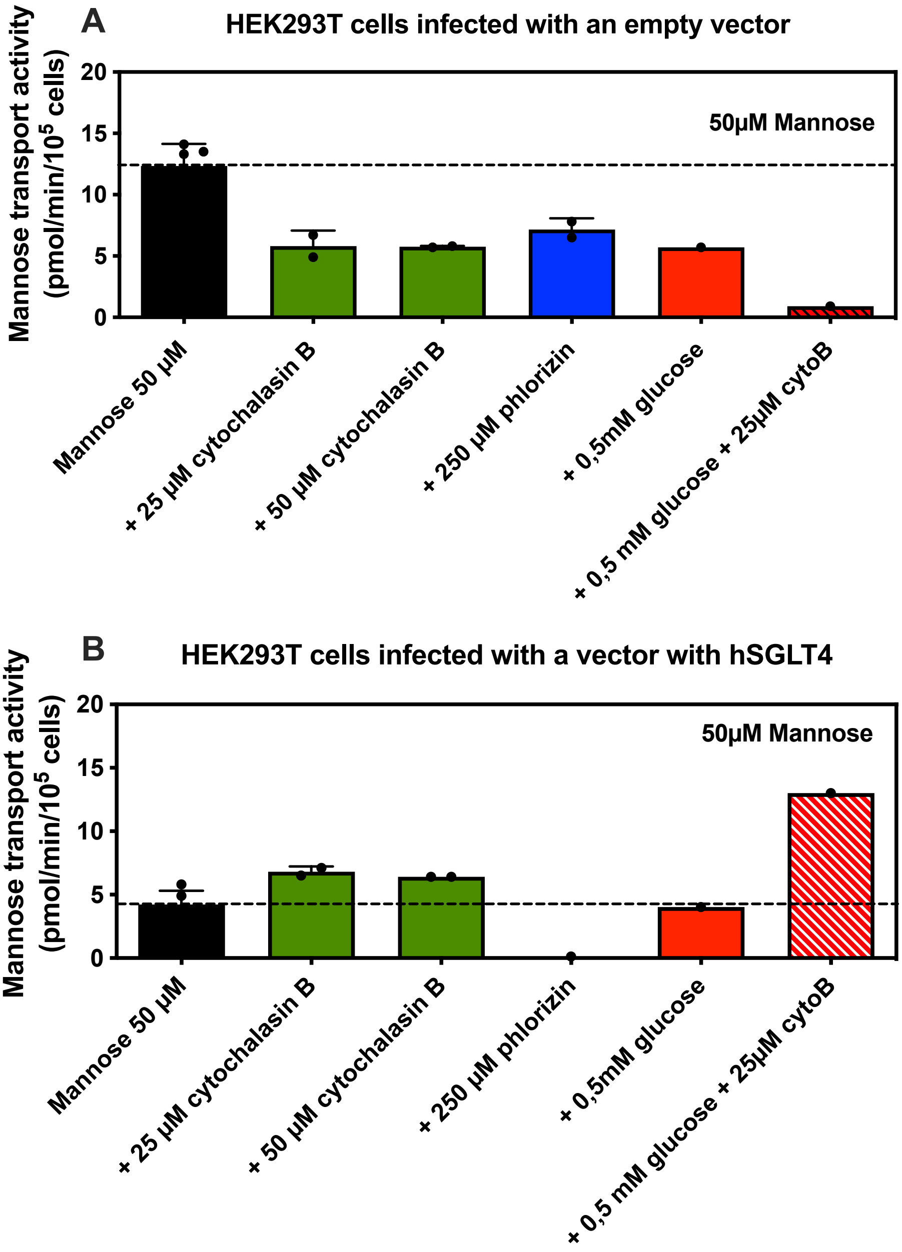

Supplementary Fig. 1: Trials to decrease non-Na-dependent transport of mannose in HEK293T cells in order to optimize measuring mannose transport activity in HEK293T cells overexpressing SGLT4.

The inhibition of 50µM U-[^{12}C+^{14}C]-mannose transport was tested in the presence of the indicated compounds and concentrations for 60 minutes at 37°C in 5% CO_2 in the in HEK293T cells transfected with and empty vector – "blank" (A) or overexpressing the active recombinant human SGLT4 transporter under the control of the CMV promoter (B). (B) The Y axis shows the specific (Na-dependent) 50µM U-[^{12}C+^{14}C]-mannose uptake due to hSGLT4 overexpression calculated by subtracting a "blank" value corresponding to the respective monosaccharide uptake obtained for HEK293T cells that were transfected with an empty plasmid and analyzed in parallel (A).

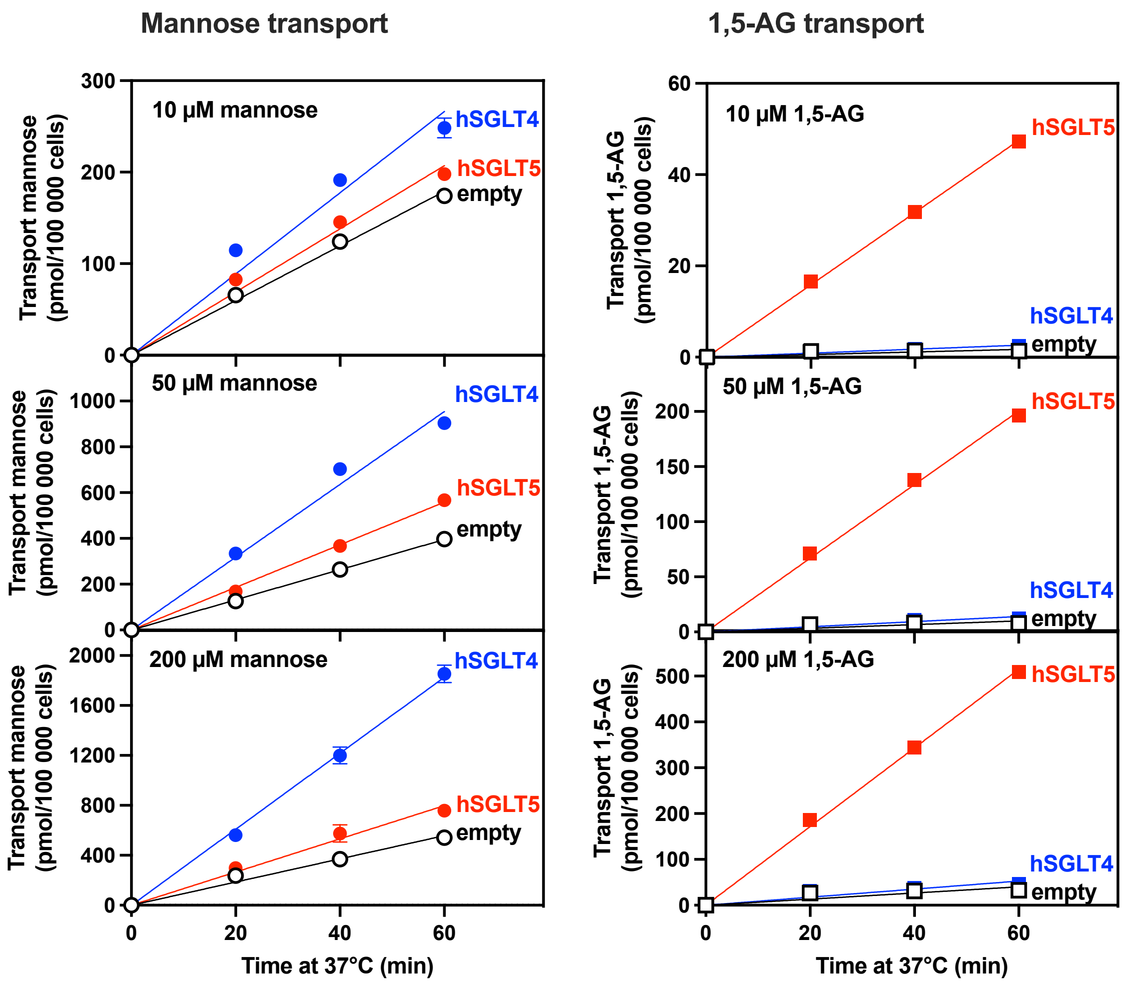

Supplementary Fig. 2: Linearity of 1,5-anhydroglucitol and mannose transport by hSGLT4 and hSGLT5-iso2.

HEK293T cells stably overexpressed hSGLT4 and hSGLT5- iso2 under the control of the CMV promoter. Transport was measured in 24 well plates with 0.6x10^6 cells during 30 and 60 mins at 37°C in 5% CO_2, in the presence of the indicated concentrations of 2-[^{1}H+^{3}H]-1,5-AG (right panel) or U-[^{12}C+^{14}C]-mannose (left panel). The transport is also shown for the control "blank cells" that were transfected with an empty plasmid carrying the CMV promoter. Data corresponds to n=3 of least two independent experiments.

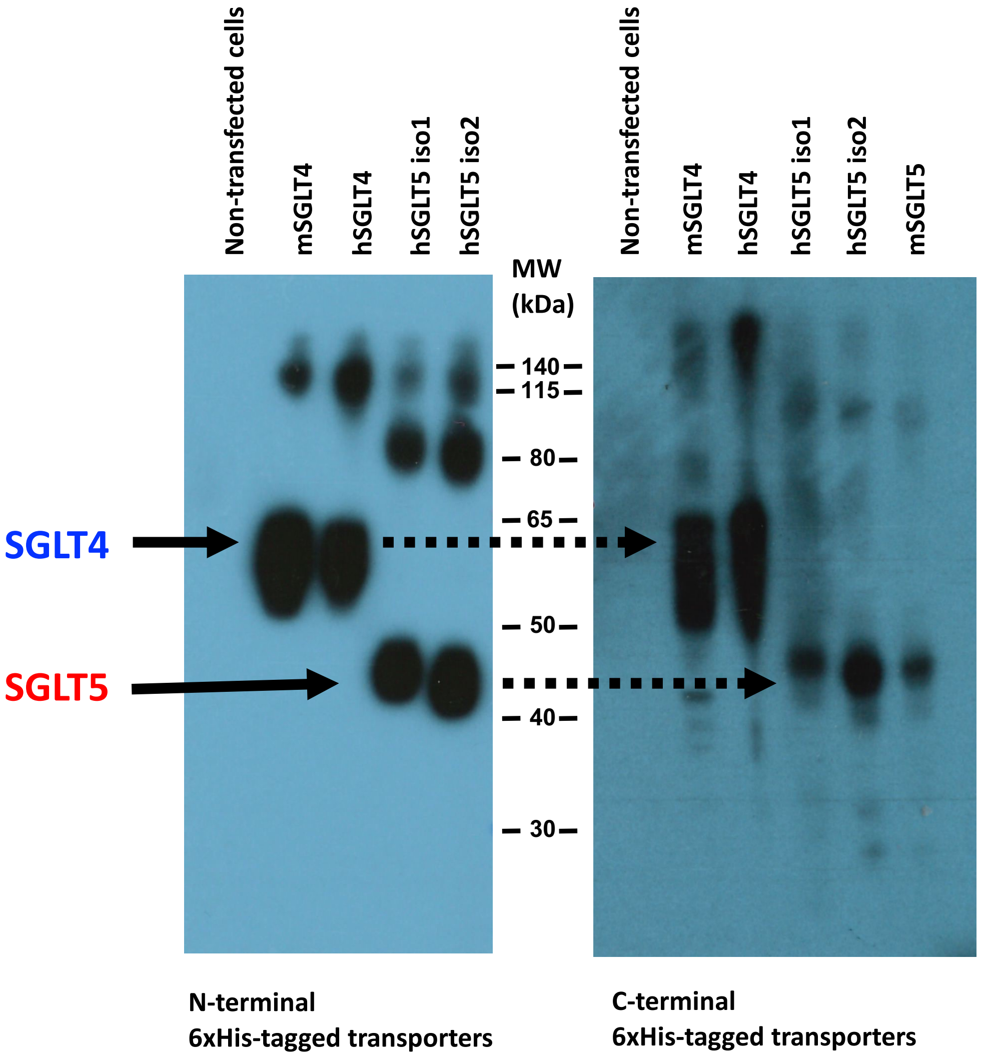

Supplementary Fig. 3: Western blot analysis of mouse and human SGLT4 and SGLT5 isoforms fused with a 6xHis-tag expressed in HEK293T cells.

HEK293T protein cell extracts prepared from cells transfected to transiently express the shown SGLT4 (human 74 kDa; mouse 75.06 kDa) or SGLT5 (human 64.3 kDa; mouse 64.7 kDa) shown isoforms fused with a 6xHis-tag at the N-or C-terminal ends and analysed by Western blots using primary antibodies that specifically recognized the His-tag at the N- or the C-terminal ends as described in Materials and Methods.

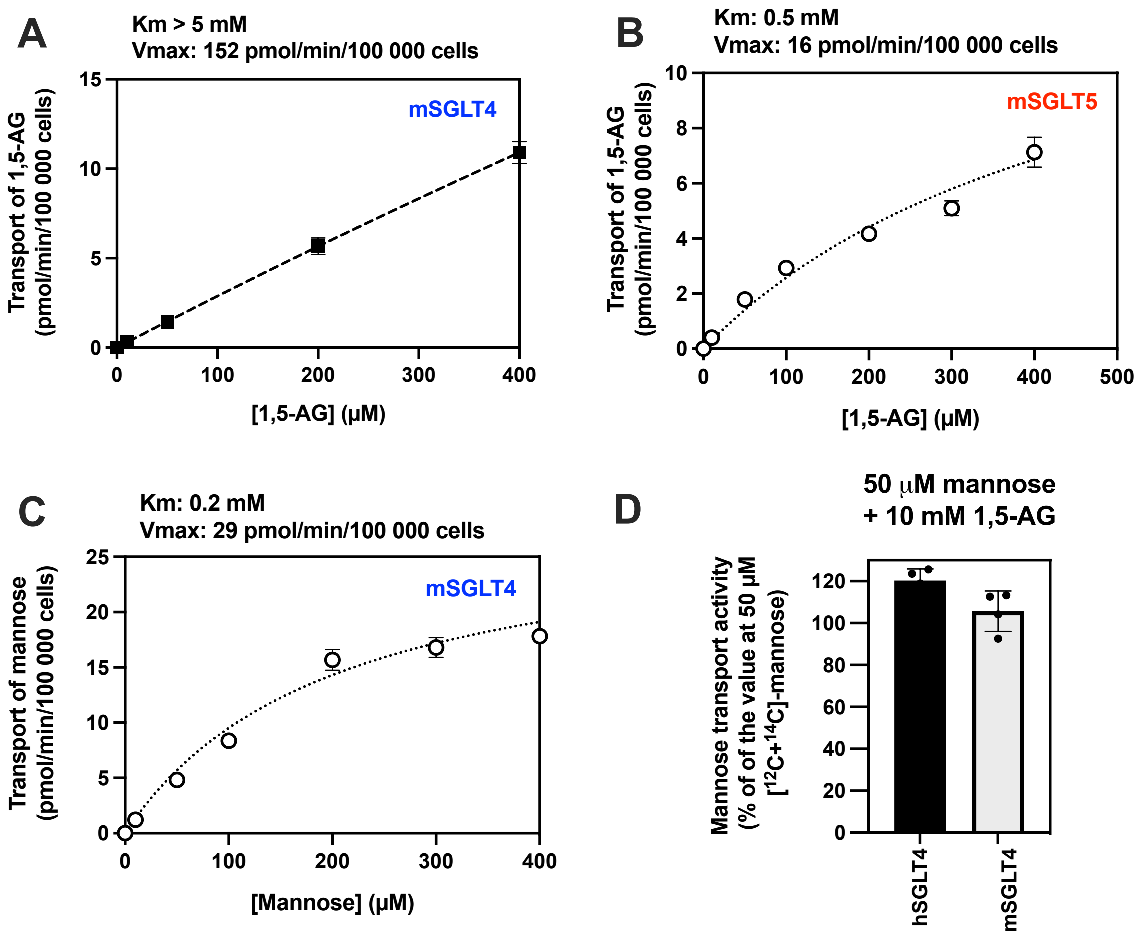

Supplementary Fig. 4: Transport activities for 1,5-anhydroglucitol and mannose of recombinant mouse SGLT4 and SGLT5 isoforms measured in HEK293T cells.

Transport was measured in 24 well plates with 0.6×10^6 cells during 30 mins at 37°C in 5% CO_2 in the presence of the indicated concentrations of 2-[^{1}H+^{3}H]-1,5-AG for both mSGLT4 (A) and mSGLT5 (B) and U-[^{12}C+^{14}C]-mannose (C) for mSGLT4. The saturation curves for the transport activities and the derived kinetic constants (Km and Vmax) are shown and indicate that mSGLT4 appears to be able to transport 1,5-anhydroglucitol but with a very low affinity (Km > 5 mM – panel A) compared to mannose (Km = 0.2 mM – panel C), while mSGLT5 transports efficiently 1,5-anhydroglucitol (Km = 0.5 mM – panel B). (D) 10 mM 1,5-anhydroglucitol does not inhibit differently the transport of mannose (measured with 50 µM U-[^{12}C+^{14}C]-mannose) for both the human (hSGLT4) and the mouse (mSGLT4) isoforms of SGLT4, indicating that both proteins are good mannose transporters. Data corresponds to n = 3 in one experiment.

```
hSGLT1 (hSLC5A1)     -------MDSS--TWSPKTTAVTRPVETHELIRNAADISIIVIYFVVVMAVGLWAMFSTN     51
hSGLT2 (hSLC5A2)     -------MEEH--T---EAGSAPEMGAQKALIDNPADILVIAAYFLLVIGVGLWSMCRTN     48
hSGLT4 (hSLC5A9)     MSKELAAMGPGASGDGVRTETAPHIALDSRVGLHAYDISVVVIYFVFVIAVGIWSSIRAS     60
hSGLT5 (hSLC5A10)    -------MAANST----------SDLHTPGTQLSVADIIVITVYFALNVAVGIWSSCRAS     43
hSMIT1 (hSLC5A3)     -----------------------------MRAVLDTADIAIVALYFILVMCIGFFAMWKSN     32
hSMIT2 (hSLC5A11)    -------MESG--TSSPQPPQLDPLDAFPQKGLEPGDIAVLVLYFLFVLAVGLWSTVKTK     51

                                             SGLT1/2:Asn78/75;His83/80    SGLT1/2:Glu102/99
                                             SGLT5:Ser70;Leu75            SGLT5:Glu94
hSGLT1 (hSLC5A1)     RGTVGGFFLAGRSMVWWPIGASLFASNIGSGHFVGLAGTGAASGIAIGGFEWNALVLVVV     111
hSGLT2 (hSLC5A2)     RGTVGGYFLAGRSMVWWPVGASLFASNIGSGHFVGLAGTGAASGLAVAGFEWNALFVVLL     108
hSGLT4 (hSLC5A9)     RGTIGGYFLAGRSMSWWPIGASLMSSNVGSGLFIGLAGTGAAGGLAVGGFEWNATWLLLA     120
hSGLT5 (hSLC5A10)    RNTVNGYFLAGRDMTWWPIGASLFASSEGSGLFIGLAGSGAAGGLAVAGFEWNATYVLLA     103
hSMIT1 (hSLC5A3)     RSTVSGYFLAGRSMTWVAIGASLFVSNIGSEHFIGLAGSGAASGFAVGAWEFNALLLLQL     92
hSMIT2 (hSLC5A11)    RDTVKGYFLAGGDMVWWPVGASLFASNVGSGHFIGLAGSGAATGISVSAYELNGLFSVLM     111
                                                                          SGLT5:Asn96

hSGLT1 (hSLC5A1)     LGWLFVPIYIKAGVVTMPEYLRKRFGGQRIQVYLSLLSLLLYIFTKISADIFSGAIFINL     171
hSGLT2 (hSLC5A2)     LGWLFAPVYLTAGVITMPQYLRKRFGGRRIRLYLSVLSLFLYIFTKISVDMFSGAVFIQQ     168
hSGLT4 (hSLC5A9)     LGWVFVPVYIAAGVVTMPQYLKKRFGGQRIQVYMSVLSLILYIFTKISTDIFSGALFIQM     180
hSGLT5 (hSLC5A10)    LAWVFVPIYISSEIVTLPEYIQKRYGGQRIRMYLSVLSLLLSVFTKISLDLYAGALFVHI     163
hSMIT1 (hSLC5A3)     LGWVFIPIYIRSGVYTMPEYLSKRFGGHRIQVYFAALSLILYIFTKLSVDLYSGALFIQE     152
hSMIT2 (hSLC5A11)    LAWIFLPIYIAGQVTTMPEYLRKRFGGIRIPIILAVLYLFIYIFTKISVDMYAGAIFIQQ     171

hSGLT1 (hSLC5A1)     ALGLNLYLAIFLLLAITALYTITGGLAAVIYTDTLQTVIMLVGSLILTGFAFHEVGGYDA     231
hSGLT2 (hSLC5A2)     ALGWNIYASVIALLGITMIYTVTGGLAALMYTDTVQTFVILGGACILMGYAFHEVGGYSG     228
hSGLT4 (hSLC5A9)     ALGWNLYLSTGILLVVTAVYTIAGGLMAVIYTDALQTVIMVGGALVLMFLGFQDVGWYPG     240
hSGLT5 (hSLC5A10)    CLGWNFYLSTILTLGITALYTIAGGLAAVIYTDALQTLIMVVGAVILTIKAFDQIGGYGQ     223
hSMIT1 (hSLC5A3)     SLGWNLYVSVILLIGMTALLTVTGGLVAVIYTDTLQALLMIIGALTLMIISIMEIGGFEE     212
hSMIT2 (hSLC5A11)    SLHLDLYLAIVGLLAITAVYTVAGGLAAVIYTDALQTLIMLIGALTLMGYSFAAVGGMEG     231

hSGLT1 (hSLC5A1)     FMEKYMKAIPTIVSDG---N-TTFQEKCYTPRADSFHIFRDPLTGDLPWPGFIFGMSILT     287
hSGLT2 (hSLC5A2)     LFDKYLGAATSLTVSEDPAV-GNISSFCYRPRPDSYHLLRHPVTGDLPWPALLLGLTIVS     287
hSGLT4 (hSLC5A9)     LEQRYRQAIPNVTVP---------NTTCHLPRPDAFHILRDPVSGDIPWPGLIFGLTVLA     291
hSGLT5 (hSLC5A10)    LEAAYAQAIPSRTIA---------NTTCHLPRTDAMHMFRDPHTGDLPWTGMTFGLTIMA     274
hSMIT1 (hSLC5A3)     VKRRYMLASPDVTSILLTYNLSNTNSCNVSPKKEALKMLRNPTDEDVPWPGFILGQTPAS     272
hSMIT2 (hSLC5A11)    LKEKYFLALASNRSE---------NSSCGLPREDAFHIFRDPLTSDLPWPGVLFGMSIPS     282

                    SGLT1/2:Trp291              SGLT1/2:Lys321
                    SGLT5:Trp278                SGLT5:Lys308
hSGLT1 (hSLC5A1)     LWYWCTDQVIVQRCLSAKNMSHVKGGCILCGYLKLMPMFIMVMPGMISRILYTEKIACVV     347
hSGLT2 (hSLC5A2)     GWYWCSDQVIVQRCLAGKSLTHIKAGCILCGYLKLTPMFLMVMPGMISRILYPDEVACVV     347
hSGLT4 (hSLC5A9)     TWCWCTDQVIVQRSLSAKSLSHAKGGSVLGGYLKILPMFFIVMPGMISRALFPDEVGCVD     351
hSGLT5 (hSLC5A10)    TWYWCTDQVIVQRSLSARDLNHAKAGSILASYLKMLPMGLIIMPGMISRALFPDDVGCVV     334
hSMIT1 (hSLC5A3)     VWYWCADQVIVQRVLAAKNIAHAKGSTLMAGFLKLLPMFIIVVPGMISRILFTDDIACIN     332
hSMIT2 (hSLC5A11)    LWYWCTDQVIVQRTLAAKNLSHAKGGALMAAYLKVLPLFIMVFPGMVSRILFPDQVACAD     342

hSGLT1 (hSLC5A1)     PSECEKYCGTKVGCTNIAYPTLVVELMPNGLRGLMLSVMLASLMSSLTSIFNSASTLFTM     407
hSGLT2 (hSLC5A2)     PEVCRRVCGTEVGCSNIAYPRLVVKLMPNGLRGLMLAVMLAALMSSLASIFNSSSTLFTM     407
hSGLT4 (hSLC5A9)     PDVCQRICGARVGCSNIAYPKLVMALMPVGLRGLMIAVIMAALMSSLTSIFNSSSTLFTI     411
hSGLT5 (hSLC5A10)    PSECLRACGAEVGCSNIAYPKLVMELMPIGLRGLMIAVMLAALMSSLTSIFNSSSTLFTM     394
hSMIT1 (hSLC5A3)     PEHCMLVCGSRAGCSNIAYPRLVMKLVPVGLRGLMMAVMIAALMSDLDSIFNSASTIFTL     392
hSMIT2 (hSLC5A11)    PEICQKICSNPSGCSDIAYPKLVLELLPTGLRGLMMAVMVAALMSSLTSIFNSASTIFTM     402

                                                                          SGLT1/2:Gln457
                                                                          SGLT5:Gln444
hSGLT1 (hSLC5A1)     DIYAKVRKRASEKELMIAGRLFILVLIGISIAWVPIVQSAQSGQLFDYIQSITSYLGPPI     467
hSGLT2 (hSLC5A2)     DIYTRLRPRAGDRELLLVGRLWVVFIVVVSVAWLPVVQAAQGGQLFDYIQAVSSYLAPPV     467
hSGLT4 (hSLC5A9)     DVWQRFRRKSTEQELMVVGRVFVVFLVVISILWIPIIQSSNSGQLFDYIQAVTSYLAPPI     471
hSGLT5 (hSLC5A10)    DIWRRLRPRSGERELLLVGRLVIVALIGVSVAWIPVLQDSNSGQLFIYMQSVTSSLAPPV     454
hSMIT1 (hSLC5A3)     DVYKLIRKSASSRELMIVGRIFVAFMVVISIAWVPIIVEMQGGQMYLYIQEVADYLTPPV     452
hSMIT2 (hSLC5A11)    DLWNHLRPRASEKELMIVGRVFVLLLVLVSILWIPVVQASQGGQLFIYIQSISSYLQPPV     462
                    SGLT5:Arg401

hSGLT1 (hSLC5A1)     AAVFLLAIFWKRVNEPGAFWGLILGLLIGISRMITEFAYGTGSCMEPSNCPTIICGVHYL     527
hSGLT2 (hSLC5A2)     SAVFVLALFVPRVNEQGAFWGLIGGLLMGLARLIPEFSFGSGSCVQPSACPAFLCGVHYL     527
hSGLT4 (hSLC5A9)     TALFLLAIFCKRVTEPGAFWGLVFGLGVGLLRMILEFSYPAPACGEVDRRPAVLKDFHYL     531
hSGLT5 (hSLC5A10)    TAVFVLGVFWRRANEQGAFWGLIAGLVVGATRLVLEFLNPAPPCGEPDTRPAVLGSIHYL     514
hSMIT1 (hSLC5A3)     AALFLLAIFWKRCNEQGAFYGGMAGFVLGAVRLILAFAYRAPECDQPDNRPGFIKDIHYM     512
hSMIT2 (hSLC5A11)    AVVFIMGCFWKRTNEKGAFWGLISGLLLGLVRLVLDFIYVQPRCDQPDERPVLVKSIHYL     522
                              SGLT5:Gly471
```

```
                                SGLT5: Ala522
hSGLT1  (hSLC5A1)     YFAIILFAISFITIVVISLLTKPIPDVHLYRLCWSLRNSKEERIDLDAEE-------ENI      580
hSGLT2  (hSLC5A2)     YFAIVLFFCSGLLTLTVSLCTAPIPRKHLHRLVFSLRHSKEEREDLDADE-------QQG      580
hSGLT4  (hSLC5A9)     YFAILLCGLTAIVIVIVSLCTTPIPEEQLTRLTWWTRNCPLSELEKEAHESTPEISERPA      591
hSGLT5  (hSLC5A10)    HFAVALFALSGAVVVAGSLLTPPPQSVQIENLTWWTLAQDVPLGTKA-------------      561
hSMIT1  (hSLC5A3)     YVATGLFWVTGLITVIVSLLTPPPTKEQIRTTTFWSKKNLVVKENCSPKEEPYKMQEKSI      572
hSMIT2  (hSLC5A11)    YFSMILSTVTLITVSTVSWFTEPPSKEMVSHLTWFTRHDPVVQKEQAPPAAPLS------      576

hSGLT1  (hSLC5A1)     QEGPKE--------------------------------------TI--------------      588
hSGLT2  (hSLC5A2)     SSLPVQNGC---------PE------------------------SA--------------      593
hSGLT4  (hSLC5A9)     GECPAGGGA---------AEN-----------------------SS--------------      605
hSGLT5  (hSLC5A10)    -----GD-----------------------------------------------------      563
hSMIT1  (hSLC5A3)     LRCSENNETINHIIPNGKSEDSIKGLQPEDVNLLVTCREEGNPVASLGHSEAETPVDAYS      632
hSMIT2  (hSLC5A11)    -LTLSQNGM---------PEASSSSS----VQF-----EMVQENTS--------------      603

hSGLT1  (hSLC5A1)     --------EIETQVPEKKKGIFRRAYDLFCGLEQHG---APK--MTEEEEKAMKMKMTDT      635
hSGLT2  (hSLC5A2)     --------MEMNEPQAPAPSLFRQCLLWFCGMSRGGVGSPPP--LTQEEAAAAARRLEDI      643
hSGLT4  (hSLC5A9)     --------LGQEQPEAPSRSWGKLLWSWFCGLSGTP---EQA--LSPAEKAALEQKLTSI      652
hSGLT5  (hSLC5A10)    --------------------------------------------------------GQTP      567
hSMIT1  (hSLC5A3)     NGQAALMGEKERKKETDDGGRYWKFIDWFCGFKSKSLSKRSLRDLMEEEAV----CLQML      688
hSMIT2  (hSLC5A11)    --------KTHSCDMTPKQSKVVKAILWLCGIQEKGKEELPA---------RAEAIIVSL      646

hSGLT1  (hSLC5A1)     SEKPLWRTVLNVNGIILVTVAVFCHAYFA-       664
hSGLT2  (hSLC5A2)     SEDPSWARVVNLNALLMMAVAVFLWGFYA-       672
hSGLT4  (hSLC5A9)     EEEPLWRHVCNINAVLLLAINIFLWGYFA-       681
hSGLT5  (hSLC5A10)    QKHAFWARVCGFNAILLMCVNIFFYAYFA-       596
hSMIT1  (hSLC5A3)     EETRQVKVILNIGLFAVCSLGIFMFVYFSL       718
hSMIT2  (hSLC5A11)    EENPLVKTLLDVNLIFCVSCAIFIWGYFA-       675
```

Supplementary Fig. 5: Sequence alignment of human SGLT5 and SGLT4 with other human transporters of the same family.

Homo sapiens sodium/glucose cotransporters SGLT1 (NP_000334.1), SGLT2 (NP_003032.1), SGLT4 (NP_001011547.2), SGLT5 (NP_001035915.1) and sodium/myo-inositol cotransporters SMIT1 (NP_008864.4) and SMIT2 (NP_001339171.1) were aligned using the Clustal Omega multiple sequence alignment program [1]. Residues highlighted in green indicate the amino acids in the substrate binding pocket (see also Fig. 4B in the main document) that direct glucose binding in SGLT1 and SGLT2 [2-4] and their conservation in the other SGLTs. Among these, the amino acids highlighted in grey are close to carbon atoms 1 and 2 of the glucose molecule and differ in SGLT5. This could explain the unique ability of SGLT5 to transport 1,5-AG. The residues highlighted in yellow show the conservation among the SGLTs of the SGLT5 variants Asn96Ile, Arg401His, Gly471Glu, that were shown to be associated with lower concentration of 1,5-AG in blood [5-8] as well as the lack of conservation among the SGLTs of Ala522 (in SGLT5), highlighted in cyan. Note that the Ala522Val is a common variant found in the population (see Supplementary Table 4) which is not associated with changes in transport activity in SGLT5 (see Fig. 5B in the main document).

```
H. sapiens          MAANSTSDLHTPGTQLSVADIIVITVYFALNVAVGIWSSCRASRNTVNGYFLAGRDMTWW      60
M. musculus         MAGNSTGDAHVPGSQLSVTDIIVISVYFALNVAVGIWSACRANKNTVSGYFLAGRDMAWW      60
G. gallus           MEGNSTAGSFTPLQQFSVADLVVIVTYFSLNLAVGIWSSCRVNRNTVSGYFLAGRDMAWW      60
X. laevis           MDRNVTSRISIPVQQFNISDIIVIAAYFLLNVAVGIWSSCRVNRNTLSGYFLAGRNMAWW      60
D. rerio            MAFNSTTKFFALSQSFSVSDIIVIGAYFLLNVAVGIWSSCRVSRNTLSGYFLAGRDMAWW      60
                    *  * *             *  **  ** ** ****** **   **  ******* * **
                         Ser70    Leu75          Glu94 Asn96Ile
H. sapiens          PIGASLFASSEGSGLFIGLAGSGAAGGLAVAGFEWNATYVLLALAWVFVPIYISSEIVTL      120
M. musculus         PIGASLFASSEGSGLFVGLAGSGAAGGLAVAGFEWNATYVLLALAWVFVPIYISSEIVTL      120
G. gallus           PIGASLFASSEGSGLFIGLAGTGAAGGIAVTGFEWNATYALLALAWVFVPVYISSGIVTM      120
X. laevis           PIGASLFASSEGSGLFIGLAGTGAAGGIAVAGFEWNATYILLALAWIFVPVYISSGIVTM      120
D. rerio            PIGASLFASSEGSGLFIGLAGTGAAGGIAVAGFEWNATYALLALAWVFVPVYVSSGIVTM      120
                    **************** **** ***** ** ******** ****** *** * ** ***

H. sapiens          PEYIQKRYGGQRIRMYLSVLSLLLSVFTKISLDLYAGALFVHICLGWNFYLSTILTLGIT      180
M. musculus         PEYIQKRFGGQRIRTYLSVLSLMLSVFTKISIDLYAGALFVHICLGWNFYLSTILTLAIT      180
G. gallus           PEYLQRRFGGERIRMYLSGLSLLLSIFTKISTDLYSGALFVQVCLGWNLYLSTVLMLLVT      180
X. laevis           PEYLQRRFGGERIQIYLSGLSLLLSVFTKISTDLYSGALFIQVCLGWNLYLSTVLMLVVT      180
D. rerio            PEYLGRRFGGERIRMYLSALSLLLSVFTKISTDLYSGALFVQVCLGWNLYLSTVLMLVVT      180
                    ***   * ** **  *** *** ** ***** *** ****   ***** **** * *  *

H. sapiens          ALYTIAGGLAAVIYTDALQTLIMVVGAVILTIKAFDQIGGYGQLEAAYAQAIPSRTIANT      240
M. musculus         ALYTIAGGLATVIYTDALQTIIMVVGAVILAVKAFNQIGGYEQLEAAYAQAIPSRTIPNT      240
G. gallus           GLYTIAGGLVAVIYTDALQTLIMVLGAIVLAVKAFNAIGGYSNLEEAYLKAVPSKIVPNT      240
X. laevis           ALYTIAGGLAAVIYTDALQTVVMIVGAVILTITAFNTIGGYQNLEEAYAKAIPSQIVPNT      240
D. rerio            ALYTIAGGLAAVIYTDTLQTFIMIIGAVILTITAFNKIGGYSNLESVYLQAVPSKIIPNT      240
                     ********  ***** ***  *  **  *   **  ****  **  *  * **    **
                                                         Trp278
H. sapiens          TCHLPRTDAMHMFRDPHTGDLPWTGMTFGLTIMATWYWCTDQVIVQRSLSARDLNHAKAG      300
M. musculus         TCHLPRADAMHMFRDPSTGDLPWTGMTFGLTIMATWYWCTDQVIVQRSLSARNLNHAKAG      300
G. gallus           TCHLPRADAMHLFRDPISGDLPWTGMTFGLSIMATWYWCTDQVIVQRSLSARSLSHAKAG      300
X. laevis           TCHLPRHDAMHLFRDPVSGDLPWTGMTFGLTILAAWYWCTDQVIVQRSLSAKNLSHAKAA      300
D. rerio            TCHLPRHDAMHLFRDPVHGDLPWPGMTLGLTIIATWYWCTDQVIVQRSLSAKSLSHAKGA      300
                    ****** **** ****  ***** *** ** * * ****************  * ***
                           Lys308
H. sapiens          SILASYLKMLPMGLIIMPGMISRALFPDDVGCVVPSECLRACGAEVGCSNIAYPKLVMEL      360
M. musculus         SILASYLKMLPMGLMIMPGMISRVLFPDDVGCVVPSECLRACGAEIGCSNIAYPKLVMEL      360
G. gallus           SILASYLKMLPLFVIIMPGMISRVLFPDAVACVDPEECTRVCGAAVGCSNIAYPKLVVEL      360
X. laevis           SILASYLKMLPMIFIVMPGMISRALYPDSVACVVPEVCLEVCGTEAGCSNIAYPKMVIEL      360
D. rerio            SIFASYLKLLPMMFIILPGMISRALYPDTVACVDPEECVKVCGAEVGCSNIAFPKLVIEL      360
                    ** ***** **      ****** * ** * ** *  *   **   ****** ** * **
                                                            Arg401His
H. sapiens          MPIGLRGLMIAVMLAALMSSLTSIFNSSSTLFTMDIWRRLRPRSGERELLLVGRLVIVAL      420
M. musculus         MPIGLRGLMIAVMMAALLSSLTSIFNSSSTLFTMDIWRQLRPSAGERELLLVGRLVIVVL      420
G. gallus           MPSGLRGLMIAVMMAALMSSLTSIFNSSSTLFTMDIWRRMRPGASERELLMVGRVVTVLL      420
X. laevis           MPSGLRGLMIAVMMAALMSSLTSIFNSSSTLFTMDIWRKIRKNANEKELLLVGRIVTVVL      420
D. rerio            MPSGLRGLMIAVMMAALMSSLTSIFNSSSTLFTMDIWKKYRRGASEKELLLVGRIVTVIL      420
                    ** ********** *** *******************   *    * *** *** * * *
                                           Gln444                     Gly471Glu
H. sapiens          IGVSVAWIPVLQDSNSGQLFIYMQSVTSSLAPPVTAVFVLGVFWRRANEQGAFWGLIAGL      480
M. musculus         IGVSVAWIPVLQGSNSGQLFIYMQSVTSSLAPPVTAIFILGIFWRRANEQGAFWGLMAGL      480
G. gallus           VALSVVWIPILQSSGGGQLYIYIQAVTSYLAPPVTAVFILAVFWPRANEQGAFWGLMAGL      480
X. laevis           VAISVVWIPILQSANSGQLYVYIQSVTSYLAPPVTAVFALAVFWRRANEQGAFWGLMVGL      480
D. rerio            VVISVVWIPILQSANSGQLYVYIQSVTSYLAPPVTAIFVMAVFWKRTNEAGAFWGLMVGL      480
                       ** *** **     ***  * * *** ******* *    ** * ** ******  **
                                                             Ala522Val
H. sapiens          VVGATRLVLEFLNPAPPCGEPDTRPAVLGSIHYLHFAVALFALSGAVVVAGSLLTPPPQS      540
M. musculus         VVGALRLVLEFLYPEPPCGQIDTRPAPLRSLHYLHFAIALFLLTCAVMAAGSLLSPPPQQ      540
G. gallus           ALGLARMGLELAHPTPRCGVPDRRPWLLADLHYLHFAALLCATAGAVVVGGSLMTPPPPS      540
X. laevis           VVGLVRMTLEFVYPFPRCGIPDERPSVLKDVHYLHFAIILCALTVGIVVGVSLLTEPPLQ      540
D. rerio            VVGLTRMVLEFAFPPPRCGVFDPAPSVLRSMHYLHFAIILCALTAIVVAVISLLTPPPTE      540
                      *  *  **   * * **  *  *  *   ******  *           **   **

H. sapiens          VQIENLTWWTLAQDVPLGTK----------------------------------------      560
M. musculus         RQIENLTWWTLAPNWSLGTK----------------------------------------      560
G. gallus           DRLRNHTWWSLSQEPPQHSMD------------G-----------TSQRYLDGSCAAAPD      577
X. laevis           SQVKNLTWWTVSQGRAPAEIALRKVSTRSQSTQGANHAVPTNCSPRSRSWSTLFCSSPET      600
D. rerio            EQTRNLTWWTLHNST-EREIPLQKVSTLSRRTDGCESVRRGKCVRT-----AGFCSPRPG      594
                        * ***

H. sapiens          -------AGDGQTPQKHAFWARVCGFNAILLMCVNIFFYAYFA     596
M. musculus         -------TGDGQTPQKRAFWARVCNVNAIFLMCVNIFFYAYFA     596
G. gallus           SWQTTEGLPALRDTTESPFWTRVCSINAIVLLCINIFCYAYFA     620
X. laevis           AVES-PKPLPPRSIKEDPFWARVCCINAIILICVNIFFYAYFA     642
D. rerio            RFASGTPPPIIHSTTEDPFWSRFCCVNAIILMCVNIFLYAYYA     637
                                      ** * *  *** * * *** *** *
```

Supplementary Fig. 6: Sequence alignment of SGLT5 orthologs.

Sodium/glucose cotransporters SGLT5 from *Homo sapiens* isoform 2 (NP_001035915.1), *Mus musculus* (NP_001369197.1), *Gallus gallus* (XP_001233919.2), *Xenopus laevis* (XP_041435094.1) and *Danio rerio* (XP_017211446.2) were aligned using the Clustal Omega multiple sequence alignment program[1]. Residues highlighted in green show the conservation across SGLT5 proteins in various species of the residues in the substrate binding pocket that align with those predicted to bind glucose in SGLT1 and SGLT2 (see also Supplementary Fig. 5). The residues highlighted in yellow show the conservation across species of the amino acids corresponding to the SGLT5 variants shown to be associated with lower concentration of 1,5-AG in blood in humans [5-8] (see also Fig. 5 in the main document). The residue highlighted in grey correspond to a frequent SNP (Ala522Val; rs12604020) found in SGLT5 in the gnomAD database.

Transporter	Substrate (10 µM)	INHIBITOR						
		1,5-AM 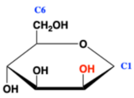	1,5-AG 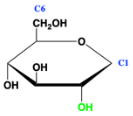	1,5-AF 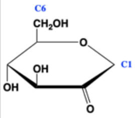	Mannose *ß-pyranose* 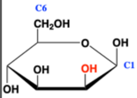	Fructose *ß-pyranose* 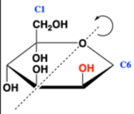	Glucose *ß-pyranose* 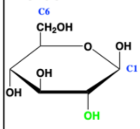	Galactose *ß-pyranose* 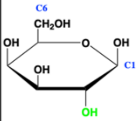
**hSGLT5-WT**	**1,5-AG**	**IC_{50} 0.013 mM**	**IC_{50} 0.167 mM**	**IC_{50} 0.179 mM**	**IC_{50} 0.380 mM**	**IC_{50} 0.726 mM**	**IC_{50} 3.65 mM**	**10 mM 10% inhibition**
**SGLT5 S70N**	**1,5-AG**	**1 mM 87% inhibition**	**IC_{50} 0.055 mM**				**2.5 mM 80% inhibition**	
**SGLT5 E71I**	**1,5-AG**	**1 mM 96% inhibition**	**IC_{50} 0.040 mM**				**2.5 mM 73% inhibition**	
**SGLT5 L75H**	**1,5-AG**	**1 mM 79% inhibition**	**IC_{50} 0.081 mM**				**2.5 mM 93% inhibition**	
**SGLT5-TM**	**1,5-AG**	**IC_{50} 3 mM**	**IC_{50} 0.033 mM**		**IC_{50} 3 mM**	**5 mM 30% inhibition**	**IC_{50} 0.132 mM**	**IC_{50} 0.172 mM**

Supplementary Fig. 7: Substrate specificity of SGLT5 - impact of replacing specific and conserved substrate binding residues in human SGLT5 by the equivalent ones in SGLT1.

When shown, the IC_{50} values are derived from inhibition curves showing the transport activity of 1,5-AG by the indicated SGLT5 mutants. Values show the concentration needed of the various sugars to inhibit by 50% the transport activity of 10 µM 2-[^{1}H+^{3}H]-1,5-AG. When the inhibition was too weak to derive IC_{50} values, the inhibition of the transport of 1,5-AG with the indicated concentration of the inhibitor-sugar is shown. Of note, despite having a lower transport activity for 1,5-AG, compared to SGLT5-WT (as shown in Fig. 4C), SGLT5-WT displayed an IC_{50} for 1,5-AG (33 µM) that is 5-fold lower than the IC50 for SGLT5-WT (162 µM). These apparently contradictory changes are not necessarily surprising: in the case of enzymes, there are multiple examples of mutations that, at the same time, increase the apparent affinity (decrease the Km) of an enzyme for its substrate while decreasing the Vmax.

Measurement of transport activities and the description of the structures of the various inhibitory sugars used are as in Fig. 3C. Data corresponds to n = 3 in at least 2 independent experiments.

Supplementary Table 1: Primers used for cloning mouse and human SGLT4 and SGLT5 in the plasmids used for their transient expression in HEK293T cells.

Start codons (ATG) and termination codons (TGA) are shown in bold. The underlined sequence before the start codon on the Fw primers corresponds to a Kozak consensus sequence that was introduced. Restriction sites introduced for cloning are highlighted in green (5' - end) and yellow (3'- end).

Fusion Protein	Sequence	Vector	Restriction site
hSGLT4-His	Fw: ATACATGAATTC<u>CACC</u>**ATG**AGCAAGGAGCTGGCAGC<br>Re: ATGTGTGCGGCCGCCACGCAAAATAGCCCCAGAG	pEF6-mycHisA	EcoRI, NotI
hSGLT4	Fw: ATACATGAATTC<u>CACC</u>**ATG**AGCAAGGAGCTGGCAGC<br>Re: ATGTGTGCGGCCGC**TCA**CGCAAAATAGCCCCAGAG	pEF6-mycHisA	EcoRI, NotI
His-hSGLT4	Fw: ATACATGAATTC<u>CACC</u>**ATG**AGCAAGGAGCTGGCAGC<br>Re: ATGTGTGCGGCCGC**TCA**CGCAAAATAGCCCCAGAG	pEF6-HisB	EcoRI, NotI
hSGLT5-His	Fw: ATACATGGTACC<u>CACC</u>**ATG**GCCGCCAACTCCACCA<br>Re: ATGTGTGCGGCCGCCAGGCGAAGTAGGCATAAAAG	pEF6-mycHisA	KpnI, NotI
HSGLT5	Fw: ATACATGGTACC<u>CACC</u>**ATG**GCCGCCAACTCCACCA<br>Re: ATGTGTGCGGCCGC**TCA**GGCGAAGTAGGCATAAAAG	pEF6-mycHisA	KpnI, NotI
His-hSGLT5	Fw: ATACATGGTACC<u>CACC</u>**ATG**GCCGCCAACTCCACCA<br>Re: ATGTGTGCGGCCGC**TCA**GGCGAAGTAGGCATAAAAG	pEF6-HisB	KpnI, NotI
mSGLT4-His	Fw: ACACATACTAGT<u>CACC</u>**ATG**AACACGGAGCTTGTGGC<br>Re: ATGTGTGCGGCCGCCACGCAAAATAGCCCCAGAG	pEF6-mycHisA	SpeI, NotI
mSGLT4	Fw: ACACATACTAGT<u>CACC</u>**ATG**AACACGGAGCTTGTGGC<br>Re: ATGTGTGCGGCCGC**TCA**CGCAAAATAGCCCCAGAG	pEF6-mycHisA	SpeI, NotI
His-mSGLT4	Fw: ACACATACTAGT<u>CACC</u>**ATG**AACACGGAGCTTGTGGC<br>Re: ATGTGTGCGGCCGC**TCA**CGCAAAATAGCCCCAGAG	pEF6-HisB	SpeI, NotI
mSGLT5-His	Fw: ACACATACTAGT<u>CACC</u>**ATG**GCTGGCAATTCCACTGG<br>Re: ATGTGTGCGGCCGCCAGGCAAAATAGGCATAGAAGA	pEF6-mycHisA	SpeI, NotI
mSGLT5	Fw: ACACATACTAGT<u>CACC</u>**ATG**GCTGGCAATTCCACTGG<br>Re: ATGTGTGCGGCCGC**TCA**GGCAAAATAGGCATAGAAGA	pEF6-mycHisA	SpeI, NotI
His-mSGLT5	Fw: ACACATACTAGT<u>CACC</u>**ATG**GCTGGCAATTCCACTGG<br>Re: ATGTGTGCGGCCGC**TCA**GGCAAAATAGGCATAGAAGA	pEF6-HisB	SpeI, NotI

Supplementary Table 2: Primers used for cloning mouse and human SGLT4 and SGLT5 in lentiviral vectors.

Start codons (ATG) and termination codons (TGA) are shown in bold. The underlined sequence before the start codon on the Fw primers corresponds to a Kozak consensus sequence that was introduced. Restriction sites introduced for cloning are highlighted in grey (5' - end) and yellow (3'- end).

Transporter (Accession nb)	Sequence	Vector	Restriction site
**hSLC5A9/hSGLT4** (NM_001011547.3/ NP_001011547.2)	Fw: ATATTCTAGACACC**ATG**AGCAAGGAGCTGGCAGC<br>Re: ACTCGAGCGGCCGC**TCA**	pUB82/pUB83	XbaI, NotI
**hSLC5A10/hSGLT5-iso1** (NM_152351.5/ NP_689564.3)	Fw: ATATTCTAGACACC**ATG**GCCGCCAACTC<br>Re: ACTCGAGCGGCCGC**TCA**	pUB82/pUB83	XbaI, NotI
**hSLC5A10/hSGLT5-iso2** (NM_001011547.3/ NP_689564.3)	Fw: ATATTCTAGACACC**ATG**GCCGCCAACTC<br>Re: ACTCGAGCGGCCGC**TCA**	pUB82/pUB83	XbaI, NotI
**mSLC5A9/mSGLT4** (NM_145551.4/ NP_663526.3)	Fw: ATATTCTAGACACC**ATG**AACACGGAGCTTGTG<br>Re: ACTCGAGCGGCCGC**TCA**	pUB82/pUB83	XbaI, NotI
**mSLC5A10/mSGLT5** (NM_001033227.2/ NP_001028399.1)	Fw: ATATTCTAGACACC**ATG**GCTGGCAATTCCA<br>Re: ACTCGAGCGGCCGC**TCA**	pUB82/pUB83	XbaI, NotI

Supplementary Table 3: Site directed mutagenesis of SGLT5-iso2.
The mutations introduced are highlighted in grey in the sequence of the primers used. For each PCR we show the plasmid that was used as a source of DNA. To obtain the plasmid coding for the SGLT5-TM, the plasmid used as a template already contained the L75H change highlighted in yellow.

**Site directed mutagenesis – SGLT5 mutants**	Sequence	Plasmid used as template
hSGLT5-S70N	Fw: 5′ CCTCTTCGCCAGCAACGAGGGCTCTGG 3′<br>Re: 3′ GGAGAAGCGGTCGTTGCTCCCGAGACC 5′	pUB83-hSGLT5-iso2
hSGLT5-E71I	Fw: 5′ CCTCTTCGCCAGCAGCATAGGCTCTGGCCTCTTCA 3′<br>Re: 3′ GGAGAAGCGGTCGTCGTATCCGAGACCGGAGAAGT 5′	pUB83-hSGLT5-iso2
hSGLT5-L75H	Fw: 5′ CGAGGGCTCTGGCCACTTCATTGGACTGG 3′<br>Re: 3′ GCTCCCGAGACCGGTGAAGTAACCTGACC 5′	pUB83-hSGLT5-iso2
hSGLT5-S70N-E71I (to create hSGLT5-TM = S70N-E71I-L75H)	Fw: 5′ CCTCCCTCTTCGCCAGCAACATAGGCTCTGGCCACTTC 3′<br>Re: 3′ GGAGGGAGAAGCGGTCGTTGTATCCGAGACCGGTGAAG 5′	pUB83-hSGLT5-L75H-iso2
**Site directed mutagenesis – SGLT5 variants**	Sequence	Plasmid used as template
hSGLT5-N96I	Fw: 5′ GGCAGGCTTCGAGTGGATTGCCACGTACG 3′<br>Re: 3′ CCGTCCGAAGCTCACCTAACGGTGCATGC 5′	pUB83-hSGLT5-iso2
hSGLT5-R401H	Fw: 5′ GAGGCGGCTGCATCCCCGCTCCG 3′<br>Re: 3′ CTCCGCCGACGTAGGGGCGAGGC 5′	pUB83-hSGLT5-iso2
hSGLT5-G471E	Fw: 5′ CCAACGAGCAGGAGGCCTTCTGGGG 3′<br>Re: 3′ GGTTGCTCGTCCTCCGGAAGACCCC 5′	pUB83-hSGLT5-iso2
hSGLT5-A522V	Fw: 5′ CTGTCGCCCTCTTTGTACTCAGTGGTGCTGT 3′<br>Re: 3′ GACAGCGGGAGAAACATGAGTCACCACGACA 5′	pUB83-hSGLT5-iso2

Supplementary Table 4: Frequency of SGLT5 inactivating sequence variations in various populations (European – non-Finish, African and South Asian).

Frequency of the sequence variations (as found in the gnomAD database) that have either been tested by transport assay (in this case the estimated residual transport activity shown as % residual activity in the column indicating the "Transport activity") or predicted to lead to lack of activity of SGLT5 (nonsense, frameshift, abnormal splicing). Of note, based on this information, more than 2 % of the Europeans – non-Finish (2.34 %) and the South Asians (2.46 %) are predicted to have low 1,5-AG. This is an underestimate, because we did not take into account the very rare inactivating mutations (total allele frequency : 0.07 %) as well as all the missense mutations that have not been experimentally tested (total allele frequency 1.13 %). Note also that at least 5 individuals (homozygous for p.Gly487Glu) are predicted to have no activity of the SGLT5 transporter. Rows showing rare variants, predicted and/or shown to decrease transport activity are highlighted in pale blue and the row showing a common variant that does not affect transport activity of 1,5-AG is highlighted in orange.

Variant ID (dbSNP)	Variant [(1)]	Effect	Transport activity	Allele frequency (x 1000)				Number of homozygotes	Allele count
				Total	European (non Finnish)	African/African American	South Asian		
rs780950637	p.Ser7AlafsTer10	frameshift	nul (pred)	0,0971	0,1573	0	0		27/278018
rs148178887	p.Asn96Ile	missense	35 % (test)	2,6571	3,6000	0,68	0,752	5	746/280742
rs142768495	c.453+2T>C	splice donor	nul (pred)	0,5630	0,4184	0,04008	0		159/282602
rs200287025	p.Gln217Ter	nonsense	nul (pred)	0,0992	0,2174	0	0	0	28/282338
rs1479130654	p.Met252ValfsTer81	frameshift	nul (pred)	0,3400	0,5978	0,08015	0,4573	0	96/282474
rs530740415	p.Tyr332Ter	frameshift	nul (pred)	0,6440	0,0000	0	5,185	0	158/245250
rs754390288	p.Arg417His (p.Arg401His)	missense	50 % (test)	0,0241	0,0089	0	0,1634	0	6/249474
rs61741107	p.Gly487Glu (p.Gly471Glu)	missense	0 % (test)	4,6297	6,7070	1,152	5,79	5	1260/ 272154
	**Total**			**9,0542**	**11,7068**	**1,9522**	**12,3477**		
rs12604020	p.Ala538Val (p.Ala522Val)	missense	110 % (test)	67,9627	20,6100	260,7	110,6	1447	19021/ 279874

[(1)] the amino acid numbering shown in brackets corresponds to the sequence of the active, shorter and more abundant transporter (hSGLT5-iso2; NP_001035915.1), while the amino acid numbering that is not in brackets is to the one used in the gnomAD database which corresponds to the longer, less abundant, not conserved and inactive isoform (hSGLT5-iso1; NP_689564.3).

Supplementary References

1 Madeira, F, Pearce, M, Tivey, ARN, Basutkar, P, Lee, J, Edbali, O, Madhusoodanan, N, Kolesnikov, A and Lopez, R. (2022) Search and sequence analysis tools services from EMBL-EBI in 2022. Nucleic Acids Res. 50, W276-279

2 Sala-Rabanal, M, Hirayama, BA, Loo, DD, Chaptal, V, Abramson, J and Wright, EM. (2012) Bridging the gap between structure and kinetics of human SGLT1. Am J Physiol Cell Physiol. 302, C1293-1305

3 Han, L, Qu, Q, Aydin, D, Panova, O, Robertson, MJ, Xu, Y, Dror, RO, Skiniotis, G and Feng, L. (2022) Structure and mechanism of the SGLT family of glucose transporters. Nature. 601, 274-279

4 Niu, Y, Liu, R, Guan, C, Zhang, Y, Chen, Z, Hoerer, S, Nar, H and Chen, L. (2022) Structural basis of inhibition of the human SGLT2-MAP17 glucose transporter. Nature. 601, 280-284

5 Li, M, Maruthur, NM, Loomis, SJ, Pietzner, M, North, KE, Mei, H, Morrison, AC, Friedrich, N, Pankow, JS, Nauck, M, Boerwinkle, E, Teumer, A, Selvin, E and Kottgen, A. (2017) Genome-wide association study of 1,5-anhydroglucitol identifies novel genetic loci linked to glucose metabolism. Sci Rep. 7, 2812

6 Loomis, SJ, Kottgen, A, Li, M, Tin, A, Coresh, J, Boerwinkle, E, Gibbs, R, Muzny, D, Pankow, J, Selvin, E and Duggal, P. (2019) Rare variants in SLC5A10 are associated with serum 1,5-anhydroglucitol (1,5-AG) in the Atherosclerosis Risk in Communities (ARIC) Study. Sci Rep. 9, 5941

7 Long, T, Hicks, M, Yu, HC, Biggs, WH, Kirkness, EF, Menni, C, Zierer, J, Small, KS, Mangino, M, Messier, H, Brewerton, S, Turpaz, Y, Perkins, BA, Evans, AM, Miller, LA, Guo, L, Caskey, CT, Schork, NJ, Garner, C, Spector, TD, Venter, JC and Telenti, A. (2017) Whole-genome sequencing identifies common-to-rare variants associated with human blood metabolites. Nat Genet. 49, 568-578

8 Boulanger, C, Stephenne, X, Diederich, J, Mounkoro, P, Chevalier, N, Ferster, A, Van Schaftingen, E and Veiga-da-Cunha, M. (2022) Successful use of empagliflozin to treat neutropenia in two G6PC3-deficient children: Impact of a mutation in SGLT5. J Inherit Metab Dis. 45, 759-768
